# The Effect of Plant Genotype, Growth Stage, and *Mycosphaerella graminicola* Strains on the Efficiency and Durability of Wheat-Induced Resistance by *Paenibacillus* sp. Strain B2

**DOI:** 10.3389/fpls.2019.00587

**Published:** 2019-05-09

**Authors:** Erika Samain, Thierry Aussenac, Sameh Selim

**Affiliations:** ^1^AGHYLE, College of Agricultural Sciences, Institut Polytechnique UniLaSalle, Beauvais, France; ^2^SDP, Laon, France; ^3^UP Transformations & Agro-Ressources, Institut Polytechnique UniLaSalle, Beauvais, France

**Keywords:** *Mycosphaerella graminicola*, *Paenibacillus* sp. strain B2, induced systemic resistance, pathogen strain, wheat genotype, wheat-growth stage

## Abstract

Plant-growth-promoting rhizobacteria are known as potential biofertilizers and plant-resistance inducers. The current work aims to study the durability of the resistance induced as a response to the inoculation of wheat grains with *Paenibacillus* sp. strain B2 (PB2) and its influence by plant genotype, growth stage, and *Mycosphaerella graminicola* strain (the causal agent of Septoria tritici blotch or STB). The results of the plate-counting method showed that PB2 has high potential for wheat-root external colonization [>10^6^ colony-forming unit (CFU)/g of root], and the quantitative real-time polymerase chain reaction (qPCR) analysis demonstrated its internal root-colonization capacity on all tested cultivars. However, the colonization seems to be dependent on wheat-growth stage. The durability of PB2-induced resistance (PB2-IR) was tested at the 3-leaf, tillering, and flag-leaf-growth stages. Additionally, the results showed that the PB2-IR is durable and able to protect the flag leaf, the most important leaf layer during grain fill. It conferred a high protection efficiency (55–94%) against four virulent strains of *M. graminicola* and over 11 wheat cultivars with different resistance levels to STB. Although, PB2-IR is dependent on *M. graminicola* strains, wheat genotypes and growth stages, its efficiency, under field conditions, at protecting the last wheat-leaf layers was not an influence. However, it showed 71–79% of protection and reached 81–94% in association with half of the recommended dose of Cherokee^®^ fungicide. This may be explained using laboratory results by its direct impact on *M. graminicola* strains in these leaf layers and by the indirect reduction of the inoculum coming from leaves infected during the earlier growth stages. Gene expression results showed that PB2-IR is correlated to upregulation of genes involved in defense and cell rescue and a priming effect in the basal defense, jasmonic acid signaling, phenylpropanoids and phytoalexins, and reactive oxygen species gene markers. To conclude, PB2 induces a high and durable resistance against *M. graminicola* under controlled and field conditions. The PB2-IR is a pathogen strain and is plant-growth-stage and genotype dependent. These results highlight the importance of taking into consideration these factors so as to avoid losing the effectiveness of induced resistance under field conditions.

## Introduction

Septoria tritici blotch (STB), caused by *Zymoseptoria tritici* (Teleomorph: *Mycosphaerella graminicola*), is regarded as the most important disease in wheat in Europe and many other countries; yield damage from this disease can reach 40% (Selim et al., 2011). To control this pathogen, the use of fungicides, mainly the sterol 14α-demethylase inhibitors (DMI) alone or mixed with succinate dehydrogenase inhibitors (SDHI), is privileged. In addition to its negative impact on the environment, animals, and human health, its application is fast, limited only by the emergence of fungicide-resistant genotypes (Selim, 2009). However, many alternatives to the integrated management of STB and other crop diseases are studied as the resistance inducers (RIs) of plant defense mechanisms such as elicitors and plant-growth-promoting rhizobacteria (PGPR) (Selim et al., 2010; Samain et al., 2017). PGPR induce local and systemic resistance, which is commonly known as induced systemic resistance (ISR). PGPR-mediated ISR resembles pathogen-induced systemic-acquired resistance (SAR) which enhances the plant’s immunity system against biotic and abiotic stresses (van Loon et al., 1998; Samain et al., 2017). However, ISR is a quantitative non-specific resistance and broad-spectrum pathogen that is likely influenced by many factors such as environmental conditions, crop nutrition, microbial communities, pathogen strain, and host genotype (Walters et al., 2005; Samain et al., 2017). Unfortunately, almost all investigations under controlled conditions are realized with a limited number of strains and plant genotypes and even at the early plant-growth stage. This could explain why the high efficiency of the resistance induced under laboratory conditions is not maintained under field conditions.

Induced systemic resistance and SAR depend on different signaling pathways. Whereas, SAR acts through a salicylic acid (SA) pathway, followed by upregulation of pathogenesis-related (PR) genes such as *PR1, PR2*, and *PR5*, ISR depends on jasmonic acid (JA) and ethylene (ET) pathways (van Loon et al., 1998). PGPR-mediated ISR enhances the formation of physical barriers such as callose and lignin, the synthesis of plant defense chemicals as reactive oxygen species (ROS), phytoalexins and phenolic compounds (Benhamou, 1996; Samain et al., 2017), but certain PGPR do not induce PR proteins (Hoffland et al., 1995). On the other hand, the upregulation of genes, known as SAR pathway markers, such as *PR2, chitinases* and *PR1*, have been observed with PGPR including the genera *Bacillus, Pseudomonas, Paenibacillus*, and *Paraburkholderia* (Park and Kloepper, 2007; Samain et al., 2017; Baccari et al., 2019). The overexpression of *PR1* induced by PGPR might be explained by the abiotic stress as a response to root colonization by bacteria (Timmusk and Wagner, 1999). Furthermore, the stimulation of both ISR and SAR at the same time as the simultaneous enhancement of the SA, JA, and ethylene pathways has been demonstrated without antagonism effects (van Wees et al., 2000). On this subject, we previously showed that *Paenibacillus* sp. strain B2 (PB2) protects wheat plants against STB, and this was associated with upregulation of *PR1* and other genes implicated in the basal defenses, ROS, SA, and JA pathways. However, these results were wheat-genotype dependent (Samain et al., 2017).

Moreover, PB2 produces the cyclic lipo-polypeptides paenimyxin antibiotics, which are antagonistic to several plant-pathogenic bacteria (Gram + and Gram -) and fungi, including *M. graminicola* (Selim et al., 2005; Samain et al., 2017), and have no negative effects on the genetic structure of soil microbial communities (Selim et al., 2007). Moreover, paenimyxin induces resistance in alfalfa (*Medicago truncatula*) against *Fusarium acuminatum* and in wheat against *M. graminicola* (Selim et al., 2010; Samain et al., 2017).

On the other hand, it has been demonstrated that PB2 promotes plant growth only in co-inoculation with another beneficial microorganism known as *Funneliformis mosseae* (previously *Glomus mosseae*) in tomato (Budi et al., 1999) and *Curtobacterium plantarum* in wheat (Samain et al., 2017), by its helper effect stimulating root mycorrhization and colonization, respectively.

The objectives of the current work are to study the durability and efficiency of the resistance induced by PB2 against STB over different wheat genotypes, growth stages and pathogen strains.

## Materials and Methods

The experimental design of this work (Supplementary Figure S1) covered the study of: (1) the impact of wheat genotypes and growth stage on the colonization of roots by PB2, (2) the impact of wheat genotypes on the resistance induced by PB2 against STB, (3) the impact of wheat-genotype-growth-stage–*M. graminicola* strain interactions on durability of the resistance induced by PB2, (4) gene expression analysis of PB2-wheat-genotype–*M. graminicola* strain interaction, and (5) to confirm the PB2-resistance induced against *M. graminicola* under field conditions.

### Microorganisms and Inoculum Preparation

*Paenibacillus* sp. strain B2 (Budi et al., 1999) was kindly provided by Dr. van Tuinen, INRA Dijon, France. Four highly virulent *M. graminicola* strains were used in this study: strain IPO323 (provided by Dr. F. Suffert, INRA Grignon); strain 1193, characterized as a moderately resistant strain to DMI fungicides (TriMR), with three SNP mutations (M-281-V, A-379-G, I381-V) (Selim, 2017, NCBI GenBank database accession number KX356102); and strains TO256 (TriMr, Selim, 2009) and ST38 (low resistance to DMI fungicides, unpublished data), which were obtained from *M. graminicola* collection strains held in the authors’ laboratory. PB2 inoculum was prepared as mentioned in Selim et al. (2005) and the *M. graminicola* inocula were prepared as mentioned in Selim et al. (2014). Briefly, to prepare the final inocula, bacterial cells, and fungal spores were collected from liquid cultures by centrifugation at 2655 × *g* for 5 min at 15°C, washed twice with sterile distilled water, and then suspended in 10 mM MgSO_4_ (Sigma^®^ M-9397) containing 0.1% Tween 20 surfactant. Bacterial cells and fungal spore vitality were checked by spreading 100 mL of inoculum on Luria-Bertani (LB) or potato dextrose agar (PDA) media, respectively.

### Plant Material and Growth Conditions

We used 11 winter-wheat cultivars – Alixan, Terroir, Altigo, Expert, Chevron, Complice, Hyking, Boregar, Cellule, Fructidor, and Hyfi – with approximately the same earliness and different levels of resistance against STB, 4, 5, 5.5, 5.5, 5.5, 6, 6, 6, 6.5, 6.5, and 7, respectively (Arvalis Institut du Végétal, 2017), on a scale from 1 (fully susceptible) to 9 (fully resistant) (Table 1). The Hyking and Hyfi cultivars are hybrids. The grains were disinfected according to Samain et al. (2017), with a few modifications, as follows: incubation in a solution of oxytetracyclin, streptomycin, penicillin, and ampicillin antibiotics (100 mg/L of each) overnight to ensure a large broad-spectrum activity against bacterial strains, then suspended in 10% calcium hypochlorite solution for 10 min and washed three times in autoclaved Milli-Q water after each disinfection step. The sterilized grains were pre-germinated on 0.5% water-agar medium and incubated in darkness at 4°C for 24 h, 20°C for 48 h, and 4°C for 24 h. The germinated grains were transferred into an inoculum of PB2 adjusted to 10^6^ CFU/mL of 10 mM MgSO_4_; 1 mL per grain for 1 h with light shaking. For the non-inoculated control, the grains were immersed in 10 mM MgSO_4_. After inoculation, the grains were transferred into 250-mL pots containing a sterilized soil mixture of silt-loam soil and sand (1:1, v/v). The pots were incubated in phytotron at 18°C (±2°C), 40% humidity, for a 16-h photoperiod with 185 μmol m^-2^ s^-1^ photon flux density supplied by high-output white fluorescent tubes (Philips Master Cool White 80 W//865, Lamotte Beuvron, France). The plants were watered three times a week with 50 mL of distilled water per pot.

**Table 1:** Wheat cultivars used to study the impact of wheat genotypes on the resistance induced by *Paenibacillus* sp. strain B2.

Cultivar	Producer	Year	Susceptibility rating^∗^
Alixan	LG	2005	4
Terroir	Florimond Desprez	2013	5
Altigo	LG	2007	5.5
Expert	Syngenta	2008	5.5
Chevron	Saaten Union	2009	5.5
Complice	Florimond Desprez	2016	6
Hyking^∗∗^	Saaten Union	2016	6
Boregar	RAGT	2008	6
Cellule	Florimond Desprez	2012	6.5
Fructidor	Unisigma	2014	6.5
Hyfi^∗∗^	Saaten Union	2013	7


*^∗^The susceptibility rating is on a scale of 1–9, where 1 represents “susceptible” and 9 represents “resistant” (Arvalis Institut du Végétal, 2017). ^∗∗^Hybrid cultivars.*

### Root Colonization by PB2

External and internal root colonization by PB2 was evaluated 7 days after sowing (das) by counting CFU in LB agar medium, as mentioned in Samain et al. (2017), using the four wheat cultivars Alixan, Altigo, Cellule, and Hyfi and using specific primers of the 16S rDNA of PB2 at 21 das. External and internal colonization by PB2 was also determined at the 3-leaf (3-L) and flag-leaf (FL) growth stages (GS) on Alixan and Cellule, by the quantitative real-time PCR (qPCR). Specific primers were designed based on the PB2 16S rDNA gene (GenBank accession No AJ011687) (Table 2). DNA was extracted from the plant roots using DNeasy 96 Plant kit (Qiagen, United States), according to the manufacturer’s protocol. DNA quantity and quality were confirmed by Nanodrop (Thermo Fisher Scientific, Waltham, MA, United States). SYBR Green qPCR assays were carried out in a reaction mixture of 25 μL that contained the following: 12.5 μl Universal Quantifast SYBR Green PCR master mix (Qiagen, United States), 0.3 μM of each primer, 50 ng of DNA, and water up to a volume of 25 μl. The conditions of the quantitative PCR were as follows: 5 min at 95°C, followed by 40 cycles of 10 s at 95°C and 30 s at 60°C. One final step from 60 to 95°C with an increase of 0.2°C s^-1^ was added to obtain a specific melting curve (Table 2 and Supplementary Figure S3). All quantitative PCR was carried out using StepOnePlus (Thermo Fisher Scientific^®^).

**Table 2:** Oligonucleotide primer sequences of wheat-defense genes and *Paenibacillus* sp. strain B2 16S rDNA.

Gene name	GenBank accession N°	For/Rev primers (5′–3′)^∗∗^	Tm^∗^ (°C)	Amplicon length (bp)	MT^∗^ (°C)	PCR efficiency (%)	Primers’ reference
**Housekeeping genes**							
Glyceraldehyde-3-phosphate dehydrogenase (GAPDH)	AF251217	AAGGGCATTTTGGGTTACGTT CCTGTTGTCACCCTGGAAGTC	58.4 58.1	63	79.1	103.98	Ors et al., 2018
β-tubulin (B-TUB)	U76897	CTGCCTCCAAGGTTTCCAAGTA GTGTCCATCCCGGAACCA	59.2 58.7	63	80.9	92.70	Ors et al., 2018
**Cell wall proteins and basal defense**							
Pathogenesis-related protein (PR1)	HQ848391	CATGCACCTTCGTATGCCTAACT TGGCTAATTACGGCATTCCTTT	58.9 59.4	52	79.1	90.25	Ors et al., 2018
Chitinase (CHIT)	AB029935	GGGTGGACCTGCTGAACAAT AGAACCATATCGCCGTCTTGA	58.4 58.3	75	84.6	92.35	Ors et al., 2018
β-1,3-glucanase (GLU)	DQ090946	TCCTGGGTTCAGAACAATGTCC TTGATGTTGACAGCCGGGTAGT	59.8 60.4	50	78.2	105.35	Ors et al., 2018
Thaumatin-like protein (TLP)	CD86039	AGGTAATTTTTTTATTGCCCTGTACTG TTACAGCCGCCGTACTACATGT	58.9 60.3	89	77.7	90.25	Ors et al., 2018
**JA signaling pathway**							
Lipase (LIP)	TaBs117A2	CACAAAATATCGACCCACCAC ACTGGGTATTCGTCTGTCAGC	60 59	149	86.3	100.92	
Lipoxygenase (LOX)	U32428	GGGCACCAAGGAGTACAAGGA GCTCGTGATGGTGTGGATGA	59.9 59.1	66	82.2	99.66	Ors et al., 2018
Allene oxide synthase (AOS)	AY196004	AGGCCGGAGAGAAGTTCCAC CCGACTTGGTCAGCTCCATC	59.3 59.2	119	88.0	93.65	Ors et al., 2018
**Phenylpropanoid and phytoalexin pathway**							
Phenylalanine ammonia-lyase (PAL)	AY005474	GTCGATTGAGCGTGAGATCAAC CACGGGAGACGTCGATGAG	58.0 59.0	59	80.5	101.35	Ors et al., 2018
Chalcone synthases (CHS)	AY286097	GCGCCTGCGTACTCTTCATC CCTCGGCGGAGCGTTT	60.0 59.0	51	80.8	109.67	Ors et al., 2018
Flavonoid 7-O-methyltransferase-like (FLAV)	CA682712	GACAACAAGGAGGCTGTGTATGG GGTGTAATGCAGTTGAATCAAGGA	62.4 59.3	117	80.6	93.43	
**Reactive oxygen species (ROS)**							
Peroxidase (POX)	X85228	TGCTTTGTCCAAGGCTGTGA GACCCGCGTTTTGTTCCA	59.0 59.1	61	79.8	108.68	Ors et al., 2018
Oxalate oxidase (OXO)	AJ556991	GCCAGAACCCCGGTATCG GGTGGGTTGGAGCTGAAGAG	60.0 58.0	55	80.8	97.23	Ors et al., 2018
Glutathione-s-transferase (GST)	AF397085	CGCTCTGAGCCCCATTCTC GGCTCCCCCAAGCATAGG	59.5 59.0	55	79.1	106.28	
Germin-like-protein (GLP)	Y09916	AGGTGAGCTCCTTGTTGGAATC GTTGAACTGGAAGTGCATGAGG	60.3 60.3	121	85.6	91.99	
Glutathione peroxidase (GPX)	KM817777	GTTCAGTTTGCCTGCACTCG GTTCCACTTGATGCTGTCGC	58.1 57.9	141	83.3	94.17	
Catalase (CAT)	X94352	TTCAAGCAGGCTGGTGAGAG TTTCATGGGTGACACGAGCA	59.8 59.7	106	84.5	103.09	
Superoxide dismutase (SOD)	EF392662	TCAGGACCCTCTTGTGACCA CGGCCTCACGTTCTTGTACT	57.6 56.6	100	81.7	102.65	
**Defense and cell rescue**							
WRKY1 transcription factor (WRKY)	EU665424	TGGCGCAAGTATGGTCAGAA CAGCCCTGGTGGGTACATTT	58.9 58.4	77	79.3	101.35	
Related protein kinase (rpK)	KR611569	TTTTGTTGGGGATCCTGCGT GCTCAGGCTCCTCGTATTGG	61.2 58.5	128	81.5	99.66	
MAP kinase (WCK1)	AF079318	AGTTCGAGATCACGGCCAAGT GAAGGCGTTGGCGATCTTC	59.8 58.8	131	87.6	108.19	Sardesai et al., 2005
***Paenibacillus* sp. strain B2**							
16S ribosomal DNA (16S rDNA)	AJ011687	TCGTAAAGCTCTGTTGCCAGG CTTGAGCAGTTACTCTACAAGACGTTC	59.0 58.0	51	75.45	109.67	


*^∗^Tm, primer’s annealing temperature; MT, amplicon’s specific melting temperature. ^∗∗^Primer pairs were designed using the Primer Express^®^ program and tested for secondary structure using the AmplifX^®^ program. All used primers did not show any form of dimerization.*

The standard curve, obtained by plotting known amounts of PB2 DNA against Ct values, was used to determine the amplification efficiency (Table 2 and Supplementary Figure S5). The resulting regression equations were used to calculate the amounts of PB2 DNA in the test samples.

### PB2-Resistance Induced in Wheat Against *M. graminicola*

Grain sterilization, pre-germination, inoculation with PB2, and phytotron conditions were carried out, as mentioned above. The 11 wheat cultivars listed above were used and infected at 3-L GS with 0.5 mL/leaf of an inoculum of *M. graminicola* strain IPO323, consisting of 2 × 10^6^ conidia/mL supplemented with 0.1% of Tween 20.

Susceptible and resistant non-hybrid wheat cultivars – Alixan and Cellule, respectively – were used to evaluate the effect of pathogen strains and growth stage on the efficiency of the resistance induced by PB2. They were inoculated with the four *M. graminicola* strains at 3-L, tillering (Ti), and FL GS. The inocula of each strain were prepared as mentioned above and applied as described in Selim et al. (2014). Briefly, 21-day-old plants were infected by spraying a 2 mL *M. graminicola* inoculum (2 × 10^6^ spores/mL) over the whole plant. Controls were sprayed with a solution of 10 mM MgSO_4_ containing 0.1% Tween 20 surfactant. Five repetitions were carried out for each condition. Three control modalities were used as non-infected with *M. graminicola* and non-inoculated with PB2 (C-), inoculated with PB2 without pathogen infection (PB2), and non-inoculated with PB2 infected with pathogen (MG). Modalities inoculated with PB2 and infected with *M. graminicola* (PB2/MG) were compared to the control modalities. Seventeen days after infection with *M. graminicola*, leaves were collected and lyophilized to evaluate the protection efficiency as a response to PB2 using the qPCR. The DNA extraction was as mentioned above and the quantification of *M. graminicola* using qPCR was realized as mentioned in Selim et al. (2014). Briefly, primers and TaqMan minor groove binder probe (For: GCCTTCCTACCCCACCATGT; Rev: CCTGAATCGCGCATCGTTA; Probe: FAM-TTACGCCAAGACATTC-MGB) were used to target a 63-bp fragment of the *M. graminicola* β-tubulin specific gene (GenBank accession no. AY547264) (Bearchell et al., 2005). A TaqMan assay was carried out in 25 μL of a reaction mixture that contained 12.5 μL Universal TaqMan PCR Master Mix (Life Technologies SAS, Villebon-sur-Yvette, France), 0.3 μM of each primer, 0.2 μM probe, 200 ng DNA and water to a volume of 25 μL. The conditions of the qPCR determination were as follows: 10 min at 95°C, followed by 40 cycles of 15 s at 95°C and 1 min at 60°C. All qPCR experiments were carried out using a StepOnePlus Real-Time PCR System (Thermo Fisher Scientific^®^). qPCR analysis of the *M. graminicola* β-tubulin gene was calibrated from 10^2^ to 10^7^ copies by serial dilution of the appropriate cloned target sequence, as previously described (Selim et al., 2011).

### RNA Extraction and Relative Gene Expression Quantification by Real-Time PCR

At the 3-L GS, aerial parts of the Alixan and Cellule plants were collected at the time of infection (T0) with *M. graminicola*, at 6, 12, 24, and 48 h after infection (hai), and 3, 5, 9, and 11 days after infection (dai) to study the evolution of the defense gene expression. Samples were stored directly in liquid nitrogen. RNA extraction and cDNA synthesis were carried out using RNeasy^®^ Mini Kit and QuantiTect^®^ Reverse Transcription Kit (Qiagen, United States), respectively, following the manufacturer’s protocol. The gene expressions of 20 wheat-defense genes were studied using specific primers (Table 2). qPCR conditions were as described in Samain et al. (2017). Briefly, the Quantifast^®^ SYBR^®^ Green PCR Kit (Qiagen, United States) and the StepOnePlus Real-Time PCR Systems (Thermo Fisher Scientific^®^) were used. Amplification conditions consisted of a denaturation cycle (95°C for 5 min) and amplification and quantification cycles (95°C for 10 s, 60°C for 30 s) repeated 40 times. One final step from 60 to 95°C with an increase of 0.2°C s^-1^ was added to obtain a specific melting curve for each studied gene (Table 2 and Supplementary Figures S2, S3). The glyceraldehyde-3-phosphate dehydrogenase (GAPDH) and β-tubulin (β-TUB) housekeeping genes were used to normalize results and to determine the expression ratio for each cDNA, as described by Ors et al. (2018). Briefly, expression ratios for each cDNA were calculated for each time point, relative to control at the same time using the 2^-ΔΔCt^ described by Livak and Schmittgen (2001), where ΔΔCt = [Ct Target (Sample) – Ct Reference (Sample)] – [Ct Target (Control) – Ct Reference (Control)] and Ct Reference = geometrical mean (Ct GAPDH: Ct β-TUB). Similar amplification efficiencies ranging between 90 and 110% were checked for all the tested primers (Table 2 and Supplementary Figures S4, S5) and expression ratio values of two were considered as a minimum to be significantly different from the control.

### Confirmation of the Efficiency of the PB2-Resistance Induced Under Field Conditions

The field site was located at Nanteuil-la-Fosse, France. Based on the susceptibility to *M. graminicola*, we used the moderately susceptible cultivars Expert and Chevron, with the resistance level of 5.5 (Arvalis Institut du Végétal, 2017). At sowing, wheat grains were coated with PB2 using 1 mL per 100 g of grains of an inoculum of 5 × 10^7^ CFU/mL of water supplemented with 20% of the general commercial sugar. Trials were performed during the 2017–2018 growing season using a completely randomized block design with four replicate plots of 4 m × 12 m. Fertilization was according to plant requirements and protection against other diseases was carried out. Twenty leaves were randomly sampled from the third leaf layer (Fn-2) below the flag leaf (Fn) at Zadoks growth stage 49 (GS49) (Zadoks et al., 1974), 14 days after the date of Cherokee^®^ (375 g/L chlorothalonil+62.5 g/L propiconazole+50 g/L cyproconazole, Syngenta, France) fungicide application at the recommended dose (RD) or half the RD (HD). The efficiency of PB2 at protecting wheat leaves against STB was compared to modalities that were non-treated, non-inoculated, fungicide-treated, or inoculated with PB2 in association with treatment with a HD of fungicide. Disease evolution was determined by assessing visual symptoms and by qPCR analysis, as mentioned above. The necrotic area related to Septoria blotch was recorded and then the leaves were stored at -80°C until lyophilization.

### Statistical Analysis

At least three biological replicates and five technical replicates were used for the experiments. For all experiments, significant differences were evaluated using ANOVA followed by Tukey’s *post hoc* test (α = 0.05) and the XLSTAT^®^ statistics program (version 2014, Addinsoft, Paris, France).

## Results

### Impact of Wheat Genotypes on Root Colonization by PB2

**FIGURE 1:**
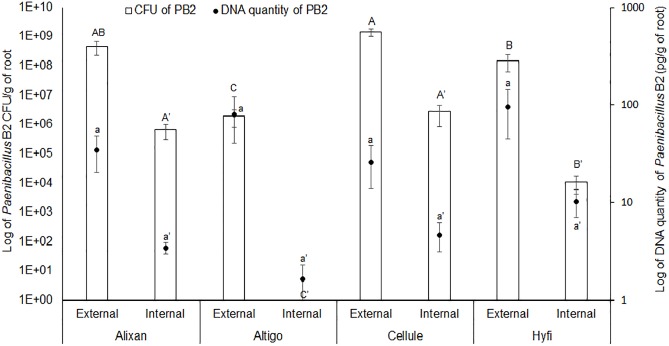
Root external and internal colonization of *Paenibacillus* sp. strain B2 (PB2) in Alixan, Altigo, Cellule, and Hyfi cultivars, which were inoculated with PB2 by immersing the pre-germinated seeds in a suspension of 10^6^ CFU/mL. The results represent the colony forming units (CFU) and PB2 DNA amount per gram of root. The values shown are the means of three biological replicates and five technical replicates. Bars indicate means ± standard deviations. Different lower-case and upper-case letters indicate significant differences between treatments, according to ANOVA followed by Tukey’s *post hoc* test (α = 0.05).

The results shown in Figure 1, of the two methods used to evaluate root colonization by PB2, confirmed the external colonization by PB2 on the four wheat cultivars. The plate method showed higher levels of root colonization in Alixan and Cellule compared to Altigo and Hyfi, whereas, qPCR analysis did not show any significant differences between cultivars. For the internal colonization, Alixan and Cellule showed higher CFU values than Hyfi, and any CFU was detected with Altigo. Using qPCR, small amounts of PB2 DNA were detected in Altigo and at the same statistical level as in the other three cultivars.

### Impacts of Wheat-Growth Stage on Root Colonization by PB2

**FIGURE 2:**
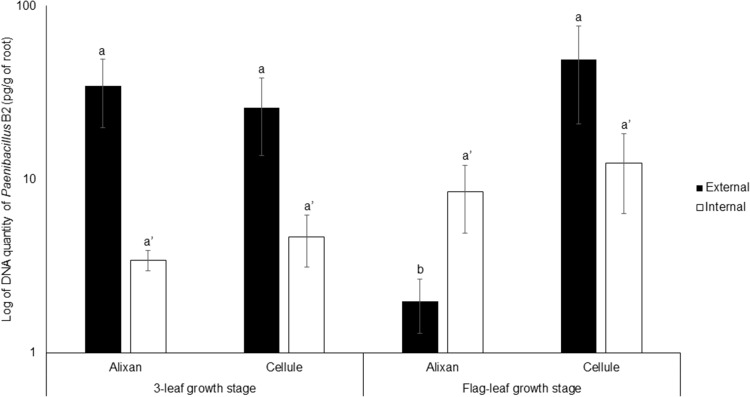
Evolution of root external and internal colonization of *Paenibacillus* sp. strain B2 (PB2) in Alixan and Cellule wheat cultivars, at 3-leaf growth stage and flag-leaf growth stage. Plants were inoculated with PB2 by immersing the pre-germinated seeds in a suspension of 10^6^ CFU/mL. The PB2 colonization, represented as DNA amount of PB2 per gram of root, was determined by qPCR. The values shown are the means of three biological replicates and five technical replicates. Bars indicate means ± standard deviations. Different lower-case letters indicate significant differences between treatments, according to ANOVA followed by Tukey’s *post hoc* test (α = 0.05).

The impact of the wheat-growth stage on root colonization by PB2 was investigated at 3-L and FL GS using the most susceptible and resistant cultivars, Alixan and Cellule, respectively, and using the qPCR method. For external root colonization with PB2, at 3-L GS, the results showed high root colonization in Alixan and Cellule, with, respectively, 34 and 26 pg of PB2 DNA/g of roots without significant differences. At FL GS, a non-significant increase in root colonization was observed in the cultivar Cellule, reaching 49 pg/g. Contrarily, a significant decrease (2 pg/g) was recorded in Alixan. Indeed, at FL GS, the external colonization of Alixan was significantly different from that of Cellule. For internal root colonization by PB2, an increase (2.5 times) was recorded in the two tested cultivars, at FL GS compared to 3-L GS (Figure 2). However, these differences were not significant between the two cultivars, nor between the two studied stages (Figure 2).

### Impacts of Wheat Genotypes on the Resistance Induced by PB2 Against STB

**FIGURE 3:**
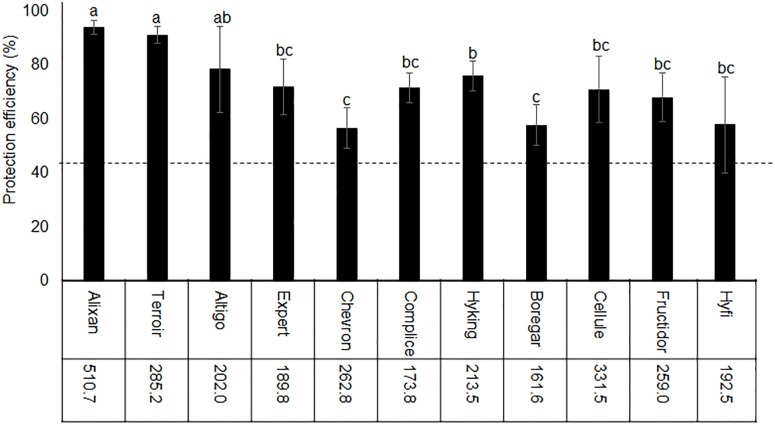
Protection efficiency induced by *Paenibacillus* sp. strain B2 (PB2) against *M. graminicola* strain IPO323 in 11 wheat cultivars with different resistance levels to this pathogen. The values shown are the means of the *M. graminicola* β-tubulin specific gene copy number in 100 ng of leaf DNA (BCN_100__ng_) in infected controls non-inoculated with PB2 of four biological replicates and five technical replicates. Bars indicate means ± standard deviations. Different lower-case letters indicate significant differences between treatments, according to ANOVA followed by Tukey’s *post hoc* test (α = 0.05).

The impact of wheat genotypes on the resistance induced by PB2 against STB was evaluated using 11 wheat cultivars with the same earliness but a different susceptibility rating given by the Arvalis Institut du Végétal. The plants had been inoculated at 3-L GS with the wild-type strain IPO323 of *M. graminicola*, and the disease level was quantified at 17 dai using qPCR and expressed in the β-tubulin copy number in 100 ng of leaf DNA (BCN_100__ng_), as previously mentioned in Selim et al. (2014). The average of leaves’ BCN_100__ng_ in the controls without PB2 were 510.7, 285.2, 202, 189.8, 262.8, 173.8, 213.5, 161.6, 331.5, 259, and 192.5, respectively, for Alixan, Terroir, Altigo, Expert, Chevron, Complice, Hyking, Boregar, Cellule, Fructidor, and Hyfi (Figure 3). The level of protection was determined as the percentage of the reduction of BCN_100__ng_ as a response to root inoculation with PB2 compared to the control infected with *M. graminicola* and non-inoculated with PB2. The results in Figure 3 show more than 56% of protection efficiency induced by PB2, over the 11 cultivars tested, with significant differences between cultivars. The two more susceptible cultivars, Alixan and Terroir, demonstrated 94 and 91% of protection, respectively; however, the protective effect induced by PB2 was not correlated with the resistance level of the cultivars.

### Impact of Wheat-Genotype-Growth-Stage–*M. graminicola* Strain Interactions on Durability of the Resistance Induced by PB2

**FIGURE 4:**
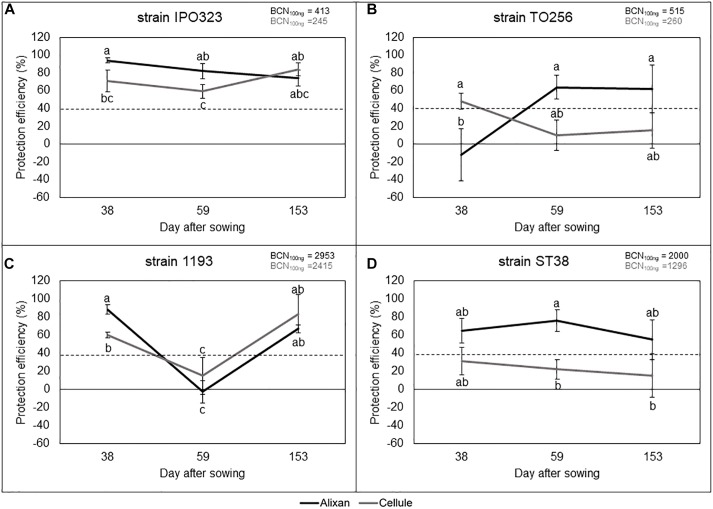
Protection efficiency induced by *Paenibacillus* sp. strain B2 against four strains of *M. graminicola*, IPO323 **(A)**, TO256 **(B)**, 1193 **(C)**, and ST38 **(D)**, in Alixan and Cellule wheat cultivars, at 38, 59, and 153 days after sowing, corresponding to the 3-leaf, tillering, and flag-leaf growth stages, respectively. Protection efficiency was determined at 17 days after infection with *M. graminicola*. The values shown are the means of four biological replicates and five technical replicates. Bars indicate means ± standard deviations. Different lower-case letters indicate significant differences between treatments, according to ANOVA followed by Tukey’s *post hoc* test (α = 0.05).

The most susceptible cultivar (Alixan) and the moderately resistant cultivar (Cellule) were used for this experiment. The resistance induced by PB2 against STB was analyzed against four *M. graminicola* strains, IPO323, 1193, ST38, and TO256, and its durability was followed from the earlier wheat-growth stages (3-L and Ti), until the FL GS, corresponding with 38, 59, and 153 das. The disease level was quantified at 17 dai using qPCR and expressed in BCN_100__ng_. The protection level against STB as a response to PB2 root inoculation was estimated by comparing it to the BCN_100__ng_ in the PB2-non-inoculated controls and we used the 40% protection level as a threshold to indicate the importance of protection as a response to root inoculation with PB2 when it is equal to, or greater than, this level (Selim et al., unpublished data). However, the global average of leaves BCN_100__ng_ of the three tested growth stages in the controls without PB2 of Alixan were 413, 515, 2950, and 2000, and of Cellule were 245, 260, 2400, and 1200 for the IPO323, TO256, 1193, and ST38 strains, respectively. Remarkably, the BCN_100__ng_ was approximately 50% less in the moderately resistant cultivar Cellule than in the susceptible cultivar Alixan, except for the strain 1193 (Figure 4A–D).

The results in Figure 4A, show high and stable protection effects against the IPO323 strain, where the BCN_100__ng_ was reduced more than 59% in the two cultivars and at the three tested growth stages. However, the protection conferred on Alixan was significantly higher than in Cellule at the two first-growth stages.

Figure 4B also shows variable protection efficiencies, as a response to PB2 root inoculation, against strain TO256 depending on cultivars and wheat-growth stages. At 3-L GS, PB2 had no protective effect on the cultivar Alixan but induced 48% of protection efficiency in Cellule (Figure 4B). At the two subsequent growth stages, this protection situation was inverted for the two cultivars, giving more than 60% of protection to Alixan and no protection to Cellule (Figure 4B). Likewise, PB2 also seemed to induce growth-stage-dependent resistance against *M. graminicola* strain 1193 with high protective effect (>60%) at the earlier and later growth stages, but it had a very low protection efficiency at the Ti GS (Figure 4C). The obtained protection efficacy was significantly higher in Alixan than Cellule at the 3-L GS (Figure 4C). Furthermore, significant differences were observed between the two cultivars at 3-L GS but not at later growth stages with strains 1193 and TO256. The protection induced by PB2 against strain ST38 was stable over the three tested growth stages and for the two cultivars (Figure 4D). Indeed, a high and stable protection efficiency (>55%) was observed for the cultivar Alixan, but with a low protection efficiency (15–30%) for Cellule (Figure 4D).

### Gene Expression Analysis of *Paenibacillus* B2-Wheat Genotype–*M. graminicola* Strain Interaction

To explain these results, the expression of genes implicated in the wheat-defense mechanisms were studied, in 3-week-old plants of the Alixan and Cellule cultivars, at time zero (T0, at the moment of infection with pathogen), and at 1 and 3 dai with *M. graminicola* IPO323 and TO256 strains. These strains were chosen to represent the two types of observed PB2-induced resistance (PB2-IR), the high and stable resistance in both cultivars against IPO323 and the growth stage cultivar-dependent resistance against the TO256 strain. Moreover, the earlier growth stage is important as the source of the inoculum for the upper-leaf layers.

**FIGURE 5:**
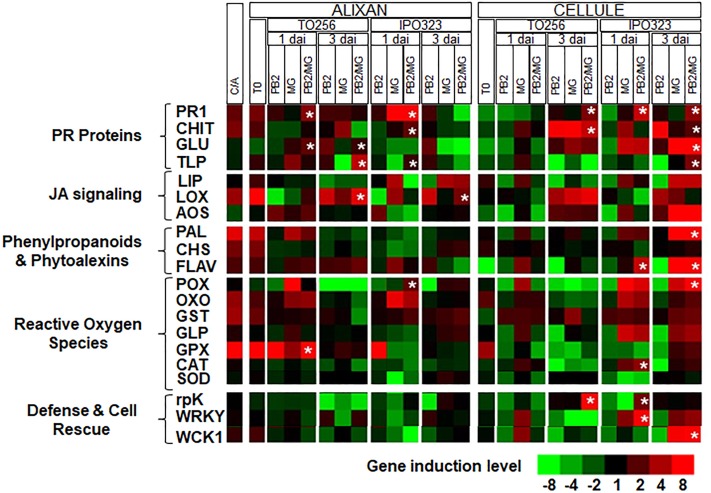
Relative expression of wheat defense genes in leaves of the moderately resistant cultivar Cellule compared to the susceptible cultivar Alixan (C/A) or as a response to root inoculation with *Paenibacillus* sp. strain B2 (PB2), leaves infected with *M. graminicola* (MG), or inoculated with PB2 and infected with MG (PB2/MG), compared to control modalities without PB2 and MG. *M. graminicola* strains IPO323 and TO256 were used. Gene expressions were studied at the 3-leaf growth stage at the moment of the leaves’ infection with *M. graminicola* (T0), 1 and 3 days after inoculation (dai). The values labeled with stars show a significant overexpression compared to MG modality. 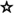 Stars indicate gene induction ≥twofolds and significant differences between PB2/MG and MG modalities, according to ANOVA followed by Tukey’s *post hoc* test (α = 0.05). The values shown are means of three biological replicates and five technical replicates.

First, the expression levels of the selected 20 defense genes were analyzed in Cellule and compared to that of Alixan in the controls (C-). The results showed that all the studied defense pathways, except these related to defense and cell rescue, and the *allene oxide synthase* (*AOS*) gene showed 1.8-fold less in Cellule compared to Alixan (Figure 5 and Supplementary Table S1).

At T0, significant upregulations (≥1.9-fold), of the *pathogenesis-related protein* (*PR1*), *chitinase* (*CHIT*), *lipase* (*LIP*), *lipoxygenase* (*LOX*), *phenylalanine ammonia-lyase* (*PAL*), *chalcone synthases* (*CHS*), *flavonoid 7-O-methyltransferase-like* (*FLAV*), *oxalate oxidase* (*OXO*), *glutathione-s-transferase* (*GST*), and *glutathione peroxidase* (*GPX*) genes, were observed in leaves of the cultivar Alixan as a response to PB2 root inoculation and 2.1- and 3.8-fold of *OXO* and *GPX* genes, respectively, in the cultivar Cellule (Figure 5 and Supplementary Table S1).

For the MG modalities, almost all of the tested genes were upregulated as infection with IPO323 and TO256 strains in both tested cultivars Alixan and Cellule. However, the genes showed more significant upregulations in the MG/PB2 modalities than in the MG modalities labeled with stars in Figure 5, and they were used to discriminate the effect of PB2 of that on that of the MG strains. Concerning the strain TO256, where a significant protection was observed in the Cellule cultivar compared to no protection in Alixan, significant upregulations of the *PR1, CHIT*, and *related protein kinase* (*rpK*) genes with 2.9, 5.0, and 9.2-fold, respectively, were observed at 3 dai of the PB2/MG modality. These genes were, respectively, 2.5, 1.6, and 7 times more than that of the MG modality (Figure 5 and Supplementary Table S1). However, none of these genes were upregulated at 1 dai. In Alixan, these genes were not upregulated at 3 dai, but at 1 dai significant upregulations were observed in the *PR1, β-1,3-glucanase* (*GLU*), and *GPX* genes with 3.5, 2.4, and 61.8-fold, respectively, and 3.5, 1.4, and 19 times more than in the MG modality.

In the second type of cultivar-independent resistance, where both cultivars showed significant protection against the IPO323 strain, in Alixan at 1 dai, the *PR1, CHIT*, thaumatin-like protein (*TLP*), and *peroxidase* (*POX*) genes were upregulated with 3.7, 2.2, 1.8, and 2.5-fold, respectively, in the PB2/MG modality, corresponding to 1.7, 1.6, 4.0, and 8.0 times, respectively, more than that of the MG IPO323 modality. At 3 dai, only the *LOX* gene showed significant upregulation with 2.8-fold in the PB2 modality, and it was not affected in the MG modality. In the PB2 modality of the Cellule cultivar at 1 dai, the *PR1, FLAV, catalase* (*CAT*), *rpK*, and *WRKY1 transcription factor* (*WRKY*) genes were upregulated with at least 2.8-fold, representing ≥1.5 times more than that of the MG modality (Figure 5 and Supplementary Table S1). At 3 dai, the overexpression of *PR1, CHIT, GLU, TLP, MAP kinase* (*WCK1*), *PAL, FLAV*, and *POX* were at least 2.0 times more than that of the MG modality (Figure 5 and Supplementary Table S1).

### Gene Expression Time-Course Analysis

**FIGURE 6:**
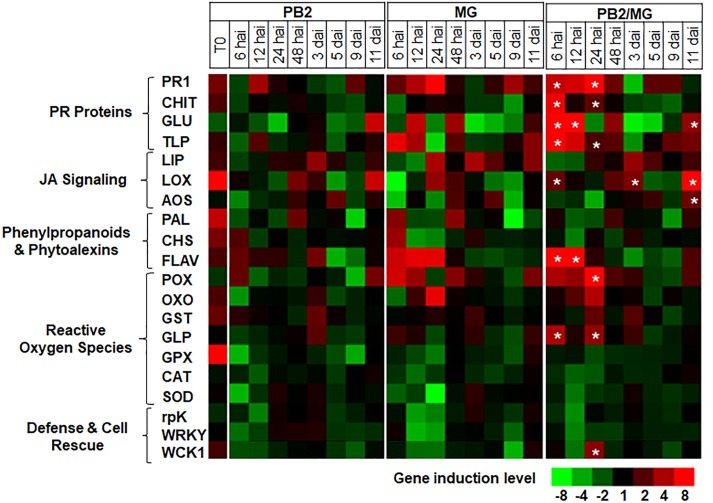
Time course of relative expression of wheat-defense genes in the susceptible cultivar Alixan at the time of infection with *M. graminicola* (MG) strain IPO323 (T0), 6, 12, 24, and 48 h after infection (hai), and 3, 5, 9, and 11 days after infection (dai). Gene expression levels of the following tested modalities, *Paenibacillus* sp. strain B2-inoculated and MG-non-infected (PB2), PB2-non-inoculated and MG-infected (MG), and PB2-inoculated and MG-infected (PB2/MG), were determined comparing to the PB2-non-inoculated and MG-non-infected control modalities. 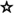 Stars indicate gene induction ≥twofolds and significant differences between PB2/MG and MG modalities, according to the ANOVA followed by Tukey’s *post hoc* test (α = 0.05). The values shown are means of three biological replicates and five technical replicates.

**FIGURE 7:**
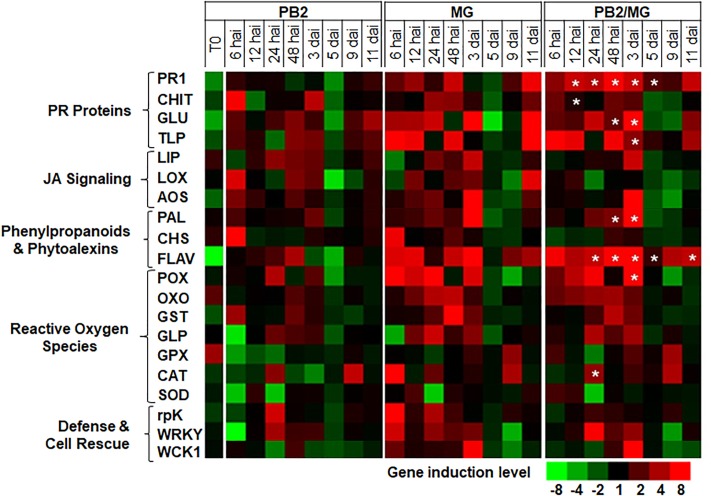
Time course of relative expression of wheat-defense genes in the moderate resistant cultivar Cellule at the time of infection with *M. graminicola* (MG) strain IPO323 (T0), 6, 12, 24, and 48 h after infection (hai), and 3, 5, 9, and 11 days after infection (dai). Gene expression levels of the following tested modalities, *Paenibacillus* sp. strain B2-inoculated and MG-non-infected (PB2), PB2-non-inoculated and MG-infected (MG), and PB2-inoculated and MG-infected (PB2/MG), were determined comparing to the PB2-non-inoculated and MG-non-infected control modalities. 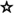 Stars indicate gene induction ≥twofolds and significant differences between PB2/MG and MG modalities, according to ANOVA followed by Tukey’s *post hoc* test (α = 0.05). The values shown are means of three biological replicates and five technical replicates.

For better understanding of the defense mechanisms implicated in wheat genotypes-*M. graminicola*-PB2 interactions, the expression of the 20 defense-related genes, as a response to root inoculation with PB2, was studied using Alixan (Figure 6 and Supplementary Table S2) and Cellule (Figure 7 and Supplementary Table S3) cultivars, at 6, 12, 24, and 48 hai, and 3, 5, 9, and 11 dai with the IPO323 strain at 3-L GS. The strain IPO323 was chosen to represent the high and stable PB2-IR in both cultivars for the objective of collecting more information about genes that could be implicated in the protection against *M. graminicola*. The genes showed a significant upregulation in the PB2/MG modalities compared to the MG modalities labeled with stars. In Alixan, this case was observed in the *PR1, CHIT, GLU, TLP, LOX, AOS, FLAV, POX*, and *germin-like-protein* (*GLP*) genes with an average expression level over studied timings of ≥2.4-fold, representing ≥2 times more than that of the MG modality (Figure 6 and Supplementary Table S2). In Cellule, the *PR1, CHIT, GLU, TLP, WCK1, PAL, FLAV, POX, CAT*, and *WRKY* genes had an overexpression average ≥2.2-fold, representing ≥1.5 times more than that of the MG modality (Figure 7 and Supplementary Table S3).

### Confirmation of the PB2-Resistance Induced Against *M. graminicola* Under Field Conditions

**FIGURE 8:**
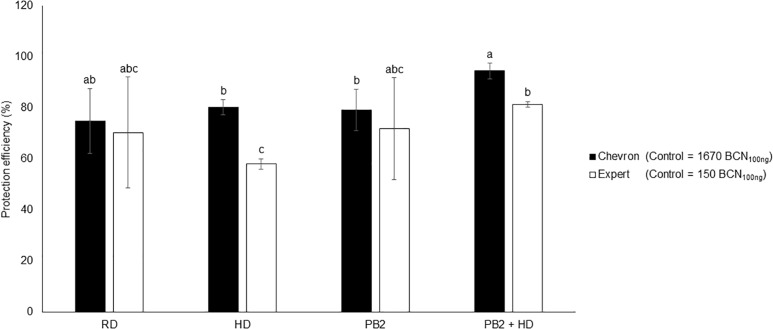
Field trials protection efficiency using the two cultivars, Expert and Chevron, as a response to wheat grains’ inoculation with *Paenibacillus* sp. strain B2 (PB2), against *M. graminicola*. The efficiency was represented as the reduction percentages of *M. graminicola* β-tubulin copy number in 100 ng of the leaf DNA (BCN_100__ng_ DNA), comparing to that in the PB2-non-inoculated and fungicide-non-treated controls. The third leaf under the flag leaf at the GS 49 was used. Cherokee^®^ fungicide, in recommended dose (RD) and half the recommended dose (HD), was used as a reference. The values shown are means of one biological replicates and five technical replicates. Bars indicate means ± standard deviations. Different lower-case letters indicate significant differences between treatments, according to ANOVA followed by Tukey’s *post hoc* test (α = 0.05).

The moderately susceptible cultivars Expert and Chevron (resistance level = 5.5) were chosen to evaluate the protection efficiency of PB2 under field conditions. The disease-infection level was determined on May 17, 2018 corresponding to the GS49 of Chevron and Expert cultivars. At this date, the STB disease pressure was very weak, and no visual symptoms were observed on the three end-leaf layers (Fn, Fn-1, and Fn-2). qPCR analysis was realized on Fn-2-extracted DNA. The results showed 80% of protection efficiency in the fungicide RD and HD modalities. The PB2 modalities showed 71 and 79% protective effects for Expert and Chevron, respectively. These protection levels increased to 81 and 94%, respectively, in Expert and Chevron in modalities where PB2 was associated with an HD of fungicide (Figure 8). As the disease pressure was still weak until the end of experimentation, the results of the yield did not show significant differences between modalities and the PB2 non-inoculated controls with an average of 10.54 and 10.96 tons/hectare in Chevron and Expert, respectively (Supplementary Figure S6).

## Discussion

With the increasing social interest in avoiding, or at least reducing, pesticide applications, the use of natural RIs as PGPRs has been one of the most important strategies studied over the last 20 years. Unfortunately, knowledge of their use is not yet sufficient to transfer laboratory results into the field in such a way as to maintain their high-protection efficiency. We showed previously that the plant genotype is one of the main factors that might influence the efficiency of RIs under field conditions (Samain et al., 2017; Ors et al., 2018). Here, we studied the impact of wheat genotypes and *M. graminicola* strains on the durability of the resistance induced over different growth stages as a response to root inoculation with *Paenibacillus* sp. strain B2.

In the current work, the protection efficiency of PB2 against STB was observed under laboratory conditions and confirmed under field conditions, where PB2 showed protection percentages similar to RD and HD of the applied fungicide. Unfortunately, the STB disease pressure was not sufficient to evaluate the impact of PB2 on yield production compared to the PB2 non-inoculated controls. On the other hand, results confirm the absence of any negative impact on the yield as a response to wheat-PB2 symbiotic relationship. Indeed, the high correlation between disease control and yield benefit usually found when fungicides are applied, is often not significant when applying RIs (Selim et al., 2010). This is probably related to the fact that plant defenses stimulation is energetically costly.

However, the laboratory results highlight the importance of factors such as *M. graminicola* strains, wheat genotypes, and growth stages in the protection induced by PB2, as well as the response of wheat-defense mechanisms to PB2-wheat genotype-*M. graminicola* strain interactions.

### Impact of Wheat Genotype and Growth Stages on Root Colonization With PB2 and the Resistance Induced Against *M. graminicola*

The results of the plate-counting method showed that root colonization with PB2 is wheat-genotype dependent. Indeed, PB2 CFU varied between cultivars external as well as internal to the roots, even though PB2 was totally absent as endophytic in the cultivar Altigo. To confirm these results, we designed specific primers that proved, using qPCR, to be highly efficient in detecting and quantifying the PB2 16S rDNA gene. On the other hand, the qPCR results confirmed the impact of wheat genotypes on the root internal amount of PB2 DNA. However, the effect of plant genotypes on the endophytic and ectophytic root colonization by PGPR was observed previously in tomato–*Pseudomonas* spp. interaction (Pillay and Nowak, 1997). This finding might be explained by the physical and chemical properties of root exudates, which vary between plant genotypes, and also by the soil microbial communities that interact during the specific dialogue between plants and PGPR (Kumar et al., 2007; Samain et al., 2017). Root exudates, which have a high diversity of organic nutriments such as sugars, vitamins, organic acids, and amino acids, represent an important source of carbon supply in the soil, attracting up to 10^10^ bacteria per gram of soil (Roesch et al., 2007; Badri and Vivanco, 2009). However, the correlation between root exudates and the endophytic PGPR is unclear (Fan et al., 2012). Furthermore, the plant root exudate composition may be modified according to plant-growth stages (Aira et al., 2010). Concerning this, our qPCR results did not show any significant effect on wheat-growth stages on the internal root colonization with PB2. Contrarily, the external root colonization by PB2 was significantly reduced at the FL GS compared to the 3-L GS and only in the Alixan cultivar. Interestingly, this reduction was not correlated with the protection conferred against STB, which maintained a higher or equal level to that of the Cellule cultivar and against all tested *M. graminicola* strains. These results indicate that the high-protection level conferred by PB2 is not proportional to the level of external colonization with PB2 but might be more correlated to internal root colonization.

### Impact of Wheat Genotype, Growth Stage, and *M. graminicola* Strain on the Durability of the Resistance Induced by PB2

The PB2-IR was influenced strongly by *M. graminicola* strains, wheat cultivars, and growth stages. Indeed, the PB2-IR was high and significant against strain IPO323 and was not dependent on either wheat genotypes or growth stages. In the case of the strain ST38, PB2-IR was influenced by wheat cultivars over all tested growth stages, since it was very low with Cellule and very high with Alixan. However, the DNA amount in the ST38 strain in the MG modality of Cellule, without root inoculation with PB2, was already 50% less than in Alixan. In fact, the populations of *M. graminicola* are qualified as highly recombinant, rapidly evolved, and locally adapted to their environment. As observed previously by Selim (2009), no significant effect of wheat cultivar was observed and the population structure was stable over all the analysis dates in the same season (Selim, 2009; Selim et al., 2014). However, under field conditions, it has never been found that there is only one genotype on the wheat-leaf samples analyzed by qPCR (Selim, 2009), and approximately two to six *M. graminicola* genotypes were determined in the same STB disease lesion (Linde et al., 2002). These observations indicate that the final protection level might be the sum of the resistance induced against the different pathogen strains. On the same hand, the impact of the wheat-growth stage was despite the case of strains 1193 and TO256. In the 1193 strain, typical responses were observed in both cultivars, where highly significant protection at 3-L and FL GS and no protection at TI GS were recorded. In the TO256 strain, no protection at 3-L GS and high protection at TI and FL GS were observed with Alixan and the opposite situation was observed with Cellule.

However, these results, as well as the results of the field trials, showed that the resistance induced is durable and maintains its high efficiency over all wheat-growth stages and especially for the last three leaf layers, which participate directly in the grain filling. The fact that PB2 controls some strains in the earlier growth stage and not in later stages, as in Cellule against strain TO256, is still indirectly important in the control of *M. graminicola* by reducing the inoculum of the upper leaf layers, which is the conidia transported from the bottom leaf layers by the impact of rain splash (Selim et al., 2014).

On the other hand, while the quantitative induced resistance mediated by PB2 is normally controlled by multiple genes and is non-specific, our results showed that it is strain-dependent and influenced strongly by plant genotype and growth stage. These results are in agreement with those reported by Ryan et al. (2010), namely that *Streptomyces scabies* isolates potato cultivars and growing season influences the resistance induced by *Streptomyces* spp. They also agree with the findings of Mazzola et al. (1995), who showed variability of protection conferred in wheat against *Gaeumannomyces graminis* var. *tritici* strains as a response to *Pseudomonas* spp.

### Defense Mechanisms Induced in Wheat as a Response to PB2–Wheat Genotype–*M. graminicola* Strain Interactions

Gene expression analysis showed that *PR1, CHIT, LOX, PAL, CHS, FLAV, OXO, GST*, and *GPX* genes were basically upregulated in Cellule cultivar compared to Alixan. These gene upregulations might explain the 50% reduction in the STB infection level in the Cellule compared to Alixan. However, gene expression results also confirmed the impact of wheat genotype-*M. graminicola* strain interaction on the resistance induced by PB2. At 3-L GS, only, the Cellule cultivar showed a high resistance to the TO256 strain as a response to PB2. This resistance was correlated to a significant upregulation at 3 dai of the *PR1, CHIT*, and *rpK* genes in the PB2/MG modality compared to the MG modality. In Alixan, where no protection was observed, almost all of the upregulated genes (*PR1, CHIT, TLP, LOX, PAL, CHS, FLAV, OXO, GST*, and *GPX*), at T0 as a response to PB2, were inhibited by the TO256 strain except the *PR1, GLU*, and *GPX* genes at 1 dai and the *GLU, TLP*, and *LOX* genes at 3 dai, which were significantly upregulated in PB2/MG modality compared to the MG modality. These results do not neglect the role of *PR1* as a protection gene marker of the wheat resistance to *M. graminicola*, as proposed previously by Adhikari et al. (2007), and highlight its possible important role in association with other defense genes. Moreover, its induction in Alixan without a protection effect against the TO256 strain may indicate that it is isolate dependent. At the same time, the protection conferred on Cellule could be related to *CHIT* and *rpK* with the possible later integration of *PR1* at 3 dai.

The importance of *CHIT* as the key to the resistance against STB has been confirmed in the second kind of resistance induced in both cultivars (Alixan and Cellule) against the IPO323 strain where it was associated with significant upregulations of *CHIT* in the PB2/MG modality compared to that of the MG modality. In addition, the *PR1, TLP*, and *POX* genes were upregulated at 1 dai and the *LOX* gene at 3 dai in Alixan, and the *PR1, FLAV, CAT, rpK*, and *WRKY* genes at 1 dai and the *PR1, GLU, TLP, WCK1, PAL, FLAV*, and *POX* genes at 3 dai in Cellule. To obtain more information about genes that could be implicated in this PB2-MG-wheat genotype interaction, we studied their expression over a time course from 6 to 11 dai, covering the biotrophic symptomless period of the infection process of *M. graminicola* until the passage into the necrotrophic phase (Selim et al., 2014). However, the results of the time course have confirmed the overexpression of genes mentioned above for both cultivars, with the *GLU, FLAV, AOS*, and *GLP* genes added for Alixan.

These results show also that the protein kinases are cultivar-dependent as the *rpK* (related protein kinase) and *WCK1* [Mitogen-Activated Protein kinase (MAPK) of wheat] genes were not upregulated in Alixan. On the other hand, they are isolate dependent, as *rpK* was upregulated in Cellule-TO256 and *WCK1* with Cellule-IPO323. It was shown that the protein kinases play important roles in promoting plant-defense reactions against biotic and abiotic stresses (Zhang et al., 1998; Ahlfors et al., 2004; Mayrose et al., 2004; Reyna and Yang, 2006). Normally, the induction of ROS and MAPK are known to occur during the earlier contact with pathogens (Tavernier et al., 1995; Pugin and Guernb, 1997; Lebrun-Garcia et al., 1998) or as a response to general elicitors, pathogen-associated molecular patterns (Kroj et al., 2003) and various fungal toxins (Rasmussen et al., 2004). As observed in the current study, the later upregulation of MAPK was observed by Rudd et al. (2008) in wheat resistance against *M. graminicola* and explained by the response to the slow growth of the pathogen within leaf; interestingly, they showed that MAPK’s activation was isolate dependent. The transcription factor *WRKY* is known by its implication in the plant tolerance to abiotic stress (Davletova et al., 2005) and also in defense against pathogens by regulating plant-defense genes directly or via association with MAPK (Pokholok, 2006).

The *CHIT* gene codes for the hydrolytic enzyme of the chitin in the pathogen cell wall, and many studies have shown its induction in resistant cultivars to *M. graminicola* contrarily to susceptible cultivars where it was downregulated (Somai-Jemmali et al., 2017; Ors et al., 2018). However, the association between the gene markers of the basal defenses, *CHIT, GLU, TLP*, and *PR1* was observed previously. The *PR-2* gene codes for the β-1,3-glucanase (GLU) also has an inhibition mode of action on the fungal development by degrading the β-1,3-glucan of the fungal cell wall and, in addition, has a signaling role in the elicitation of other plant-defense mechanisms (Ham et al., 1991; Wessels, 1993). The association of these pathogen-related proteins may be responsible for the synergistic effect on the plant defense activities against *M. graminicola*, as observed previously with *TLP* (*PR5*) (Leah et al., 1991), and the delay of the *Phaeosphaeria nodorum* incidence in wheat as a response to the combination of *CHIT* and *GLU* (Anand et al., 2003). In addition, *GLU* and *TLP* are induced in leaves by both SA and JA and exhibit glucanase activity, contrary to *GLU* alone, leading to degraded fungal plasma membrane and decreased disease incidence in potato and wheat (Vigers et al., 1992; Grenier et al., 1999; Anand et al., 2003).

Moreover, the upregulation of the *PAL* and *FLAV* genes involved in the phenylpropanoid and phytoalexin pathways, *POX, CAT*, and *GLP*, the gene markers of the ROS pathway, and *AOS* and *LOX*, the gene markers of the JA pathway, confirm our previous results concerning the association of these defense pathways with the basal-defense genes in the resistance induced in wheat against *M. graminicola* as a response to PB2 (Samain et al., 2017) where we discussed the possible defense mechanisms related to these pathways and especially against *M. graminicola*.

The upregulation of the *CAT* and *GLP* genes code for catalase and germin-like protein, respectively, indicates the plant protection against oxidative stress results in ROS pathway stimulation (Khuri et al., 2001). Catalase detoxifies H_2_O_2_, resulting in water and oxygen (Smirnoff, 1993). In addition to its role in oxygen burst detoxification, *GLP* also has protease inhibitor activity against *M. graminicola* (Segarra et al., 2003).

However, several factors may influence resistance inducer–plant–pathogen interactions, including the efficiency of the biocontrol strain, pathogen aggressiveness, host susceptibility, and environmental conditions (Francés et al., 2006; Recep et al., 2009).

## Conclusion

STB is the most economic important disease in wheat. The current work shows the efficiency of *Paenibacillus* sp. strain B2 as a biological control agent against STB, under controlled and field conditions, by inducing wheat-resistance mechanisms combined with basal defenses, ROS, phenylpropanoid and phytoalexin, SA, JA pathways, and the important role of the *chitinase* gene. This resistance is durable and able to protect the last wheat-leaf layers, which are the most important in yield production. Although PB2-induced resistance depends on *M. graminicola* strains, wheat genotypes, and growth stages, its efficiency against STB, under field conditions, is less influenced by these factors. This may be explained by its direct impact on *M. graminicola* inoculum in the upper-leaf layers, which usually consist of a mixture of genotypes, or by its indirect impact on reducing the inoculum coming from the oldest leaves infected during the earlier growth stages. Interestingly, the wheat-PB2 symbiotic relationship is not energetically costly and without negative impact on the yield production. However, results under field conditions will be confirmed during, at least, two wheat growth seasons more.

## Author Contributions

ES carried out the experimental work and wrote the manuscript. SS managed and supervised the project and the Ph.D. research programs, and revised the manuscript with TA.

## Conflict of Interest Statement

The authors declare that the research was conducted in the absence of any commercial or financial relationships that could be construed as a potential conflict of interest.

## Supplementary Material

FIGURE S1 The experimental design of this work covered the study of: (1) the impact of wheat genotypes and growth stage on the colonization of roots by PB2, (2) the impact of wheat genotypes on the resistance induced by PB2 against STB, (3) the impact of wheat-genotype-growth-stage–*M. graminicola* strain interactions on durability of the resistance induced by PB2, (4) gene expression analysis of PB2-wheat-genotype-*M. graminicola* strain interaction, and (5) to confirm the PB2-resistance induced against *M. graminicola* under field conditions. Wheat grains’ inoculation with PB2 was at sawing. Under controlled conditions, wheat leaf infection with *M. graminicola* was realized using 10^6^ spores/leaf at 3-leaf (3-L), tillering (Ti), or flag-leaf (FL) growth stage (GS). Leaf infection level was determined using quantitative real-time PCR (qPCR), at 17 days after infection. Under field conditions, leaf infection was by the natural inoculum and disease level in the third leaf under the FL was quantified using qPCR at GS 49. The highly virulent strains of *M. graminicola*, IPO323, TO256, 1193, and ST38 were used and at least two wheat cultivars with different resistant level to *M. graminicola*.

FIGURE S2 PCR’s melting curve for each primer pair used in the gene expression study. Glyceraldehyde-3-phosphate dehydrogenase (GAPDH), β-tubulin (B-TUB), pathogenesis-related protein (PR1), Chitinase (CHIT), β-1,3-glucanase (GLU), thaumatin-like protein (TLP), lipase (LIP), lipoxygenase (LOX), allene oxide synthase (AOS), phenylalanine ammonia-lyase (PAL), chalcone synthases (CHS), and flavonoid 7-O-methyltransferase-like (FLAV).

FIGURE S3 PCR’s melting curve for each primer pair used in the gene expression study and in the quantification of *Paenibacillus* strain B2. Peroxidase (POX), oxalate oxidase (OXO), glutathione-s-transferase (GST), germin-like-protein (GLP), glutathione peroxidase (GPX), catalase (CAT), superoxide dismutase (SOD), related protein kinase (rpK), WRKY1 transcription factor (WRKY), MAP kinase (WCK1), and *Paenibacillus* strain B2 16S ribosomal DNA (16S rDNA).

FIGURE S4 PCR’s amplification efficiency (E), for each primer pair used in the gene expression study, is deducted from the slopes (S) of the standard curves based on *E* = 100^∗^(10^-1/^s^-1^). Glyceraldehyde-3-phosphate dehydrogenase (GAPDH), β-tubulin (B-TUB), pathogenesis-related protein (PR1), Chitinase (CHIT), β-1,3-glucanase (GLU), thaumatin-like protein (TLP), lipase (LIP), lipoxygenase (LOX), allene oxide synthase (AOS), phenylalanine ammonia-lyase (PAL), chalcone synthases (CHS), and flavonoid 7-O-methyltransferase-like (FLAV).

FIGURE S5 PCR’s amplification efficiency (E), for each primer pair used in the gene expression study and in the quantification of *Paenibacillus* strain B2, is deducted from the slopes (S) of the standard curves based on *E* = 100^∗^(10^-1/^s^-1^). Peroxidase (POX), oxalate oxidase (OXO), glutathione-s-transferase (GST), germin-like-protein (GLP), glutathione peroxidase (GPX), catalase (CAT), superoxide dismutase (SOD), related protein kinase (rpK), WRKY1 transcription factor (WRKY), MAP kinase (WCK1), and *Paenibacillus* strain B2 16S ribosomal DNA (PB2 16S rDNA).

FIGURE S6 Field trials grain yield production the two cultivars, Expert and Chevron, as a response to wheat grains’ inoculation with *Paenibacillus* sp. strain B2, Cherokee^®^ fungicide application, in recommended dose (RD) and half the recommended dose (HD), an association between PB2 and Cherokee^®^ in HD (PB2+HD), and in PB2-non-inoculated and fungicide-non-treated controls (C-). The values shown are the means of one biological replicates and five technical replicates. Bars indicate means ± standard deviations. Different lower-case letters indicate significant differences between treatments, according to ANOVA followed by Tukey’s *post hoc* test (α = 0.05).

TABLE S1 Gene expression ratio of some wheat-defense-related genes encoding proteins from different classes, estimated by real-time PCR.

TABLE S2 Gene expression ratio in the susceptible cultivar Alixan as a response to *Paenibacillus* B2 (PB2), *M. graminicola* strain IPO323 (MG) and *Paenibacillus* B2 and *M. graminicola* strain IPO323 (PB2/MG), at the time of infection with IPO323 (T0), 6, 12, 24, and 48 h after inoculation (hai), 3, 5, 9, and 11 days after inoculation (dai).

TABLE S3 Gene expression ratio in the moderate cultivar (Cellule) as a response to *Paenibacillus* B2 (PB2), *M. graminicola* strain IPO323 (MG) and *Paenibacillus* B2 and *M. graminicola* strain IPO323 (PB2/MG), at the time of infection with IPO323 (T0), 6, 12, 24, and 48 h after inoculation (hai), 3, 5, 9, and 11 days after inoculation (dai).
