# Supplementary figures and images for: The Effect of Plant Genotype, Growth Stage, and Mycosphaerella graminicola Strains on the Efficiency and Durability of Wheat-Induced Resistance by Paenibacillus sp. Strain B2
Source: Front Plant Sci. 2019 May 9;10:587. doi: 10.3389/fpls.2019.00587 (PMC6521617; doi:10.3389/fpls.2019.00587)

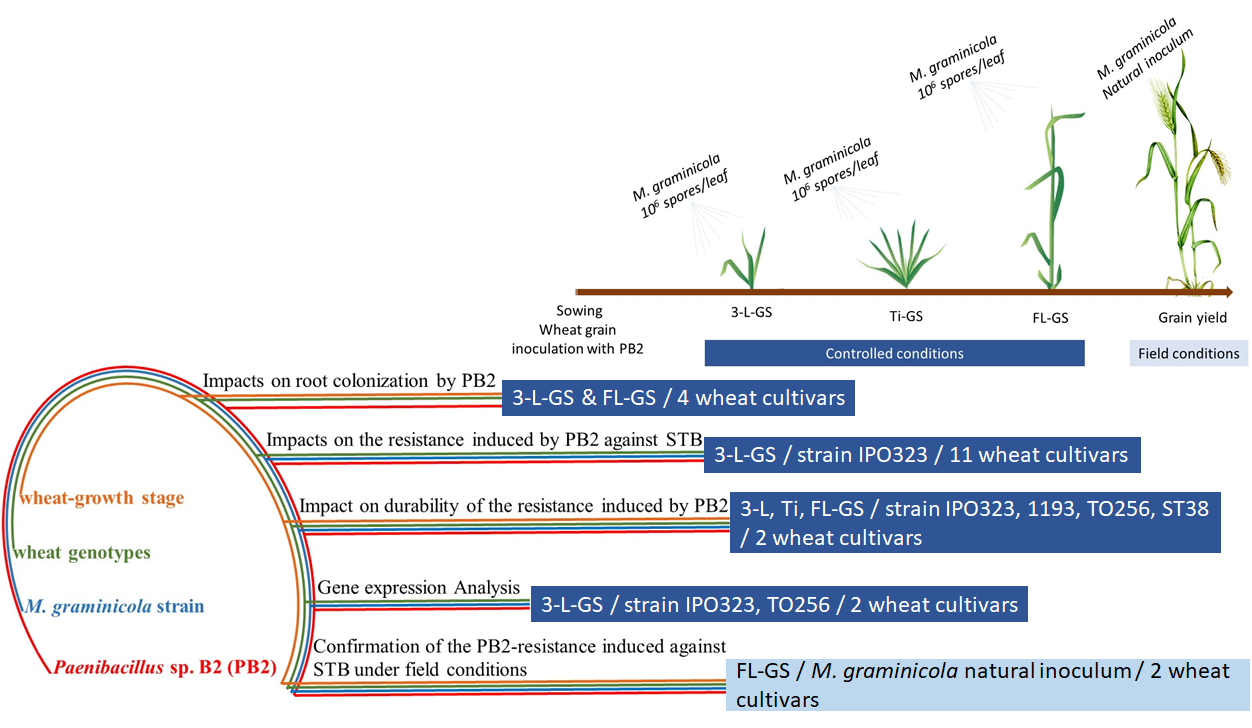

Supplement: FIGURE S1 — The experimental design of this work covered the study of: (1) the impact of wheat genotypes and growth stage on the colonization of roots by PB2, (2) the impact of wheat genotypes on the resistance induced by PB2 against STB, (3) the impact of wheat-genotype-growth-stage–M. graminicola strain interactions on durability of the resistance induced by PB2, (4) gene expression analysis of PB2-wheat-genotype-M. graminicola strain interaction, and (5) to confirm the PB2-resistance induced against M. graminicola under field conditions. Wheat grains’ inoculation with PB2 was at sawing. Under controlled conditions, wheat leaf infection with M. graminicola was realized using 106 spores/leaf at 3-leaf (3-L), tillering (Ti), or flag-leaf (FL) growth stage (GS). Leaf infection level was determined using quantitative real-time PCR (qPCR), at 17 days after infection. Under field conditions, leaf infection was by the natural inoculum and disease level in the third leaf under the FL was quantified using qPCR at GS 49. The highly virulent strains of M. graminicola, IPO323, TO256, 1193, and ST38 were used and at least two wheat cultivars with different resistant level to M. graminicola. [file Image_1.TIF]

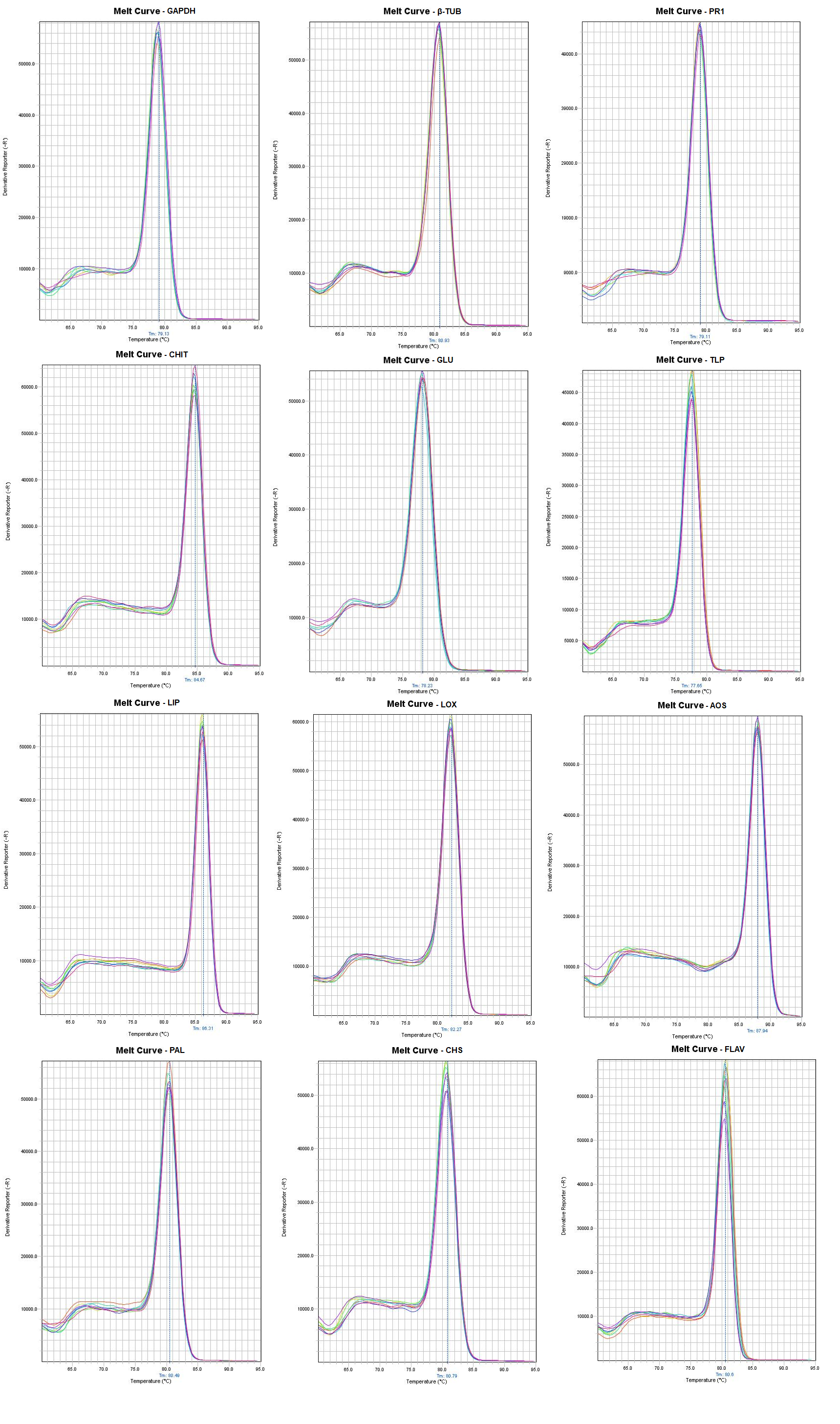

Supplement: FIGURE S2 — PCR’s melting curve for each primer pair used in the gene expression study. Glyceraldehyde-3-phosphate dehydrogenase (GAPDH), β-tubulin (B-TUB), pathogenesis-related protein (PR1), Chitinase (CHIT), β-1,3-glucanase (GLU), thaumatin-like protein (TLP), lipase (LIP), lipoxygenase (LOX), allene oxide synthase (AOS), phenylalanine ammonia-lyase (PAL), chalcone synthases (CHS), and flavonoid 7-O-methyltransferase-like (FLAV). [file Image_2.TIF]

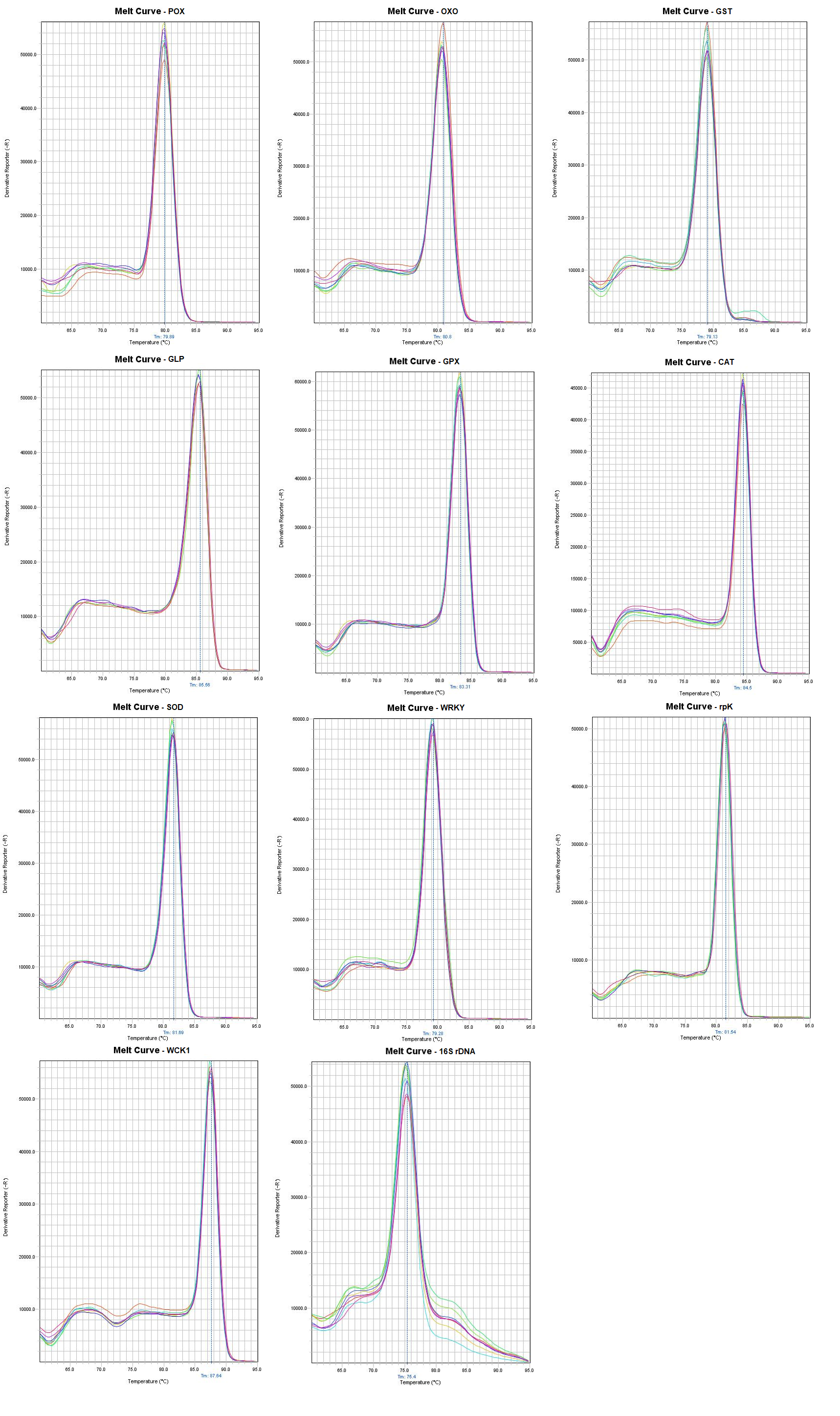

Supplement: FIGURE S3 — PCR’s melting curve for each primer pair used in the gene expression study and in the quantification of Paenibacillus strain B2. Peroxidase (POX), oxalate oxidase (OXO), glutathione-s-transferase (GST), germin-like-protein (GLP), glutathione peroxidase (GPX), catalase (CAT), superoxide dismutase (SOD), related protein kinase (rpK), WRKY1 transcription factor (WRKY), MAP kinase (WCK1), and Paenibacillus strain B2 16S ribosomal DNA (16S rDNA). [file Image_3.TIF]

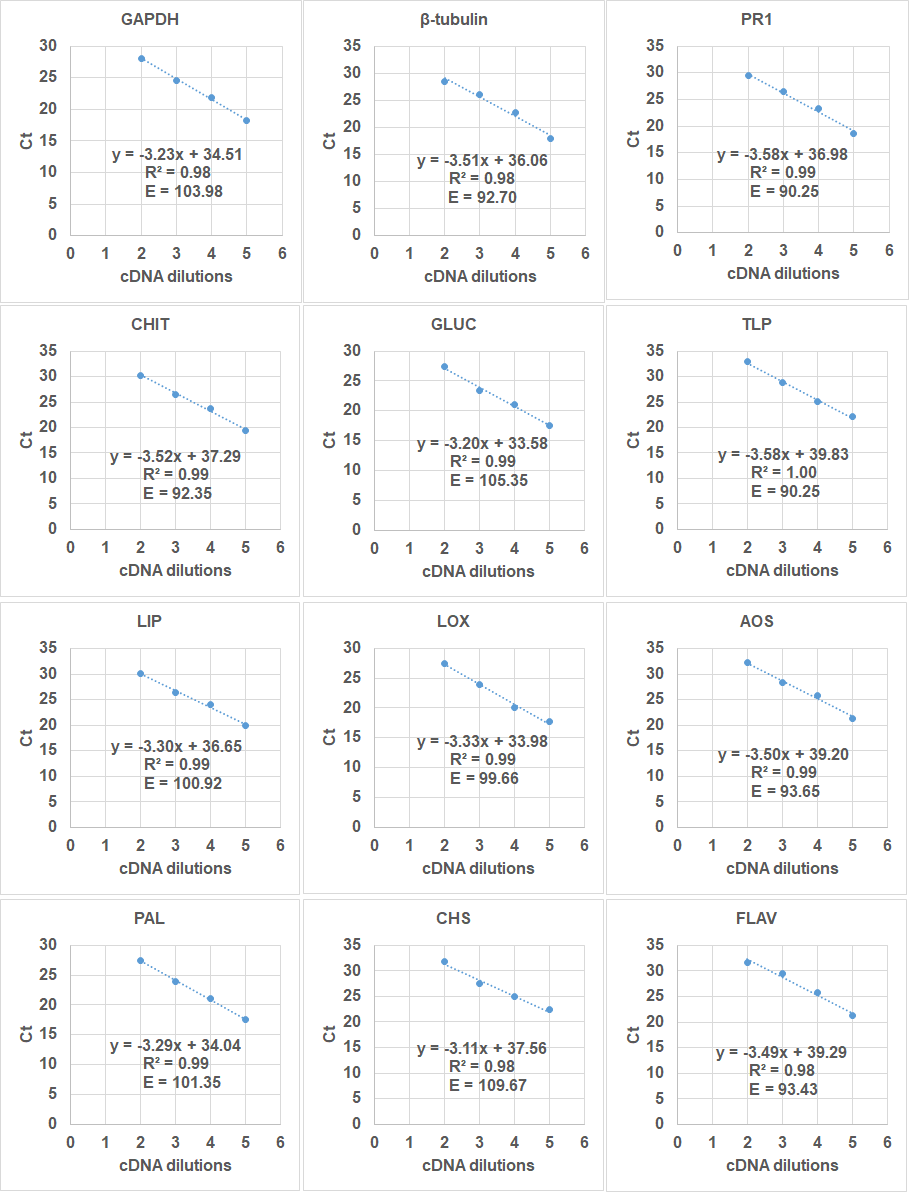

Supplement: FIGURE S4 — PCR’s amplification efficiency (E), for each primer pair used in the gene expression study, is deducted from the slopes (S) of the standard curves based on E = 100∗(10-1/s-1). Glyceraldehyde-3-phosphate dehydrogenase (GAPDH), β-tubulin (B-TUB), pathogenesis-related protein (PR1), Chitinase (CHIT), β-1,3-glucanase (GLU), thaumatin-like protein (TLP), lipase (LIP), lipoxygenase (LOX), allene oxide synthase (AOS), phenylalanine ammonia-lyase (PAL), chalcone synthases (CHS), and flavonoid 7-O-methyltransferase-like (FLAV). [file Image_4.TIF]

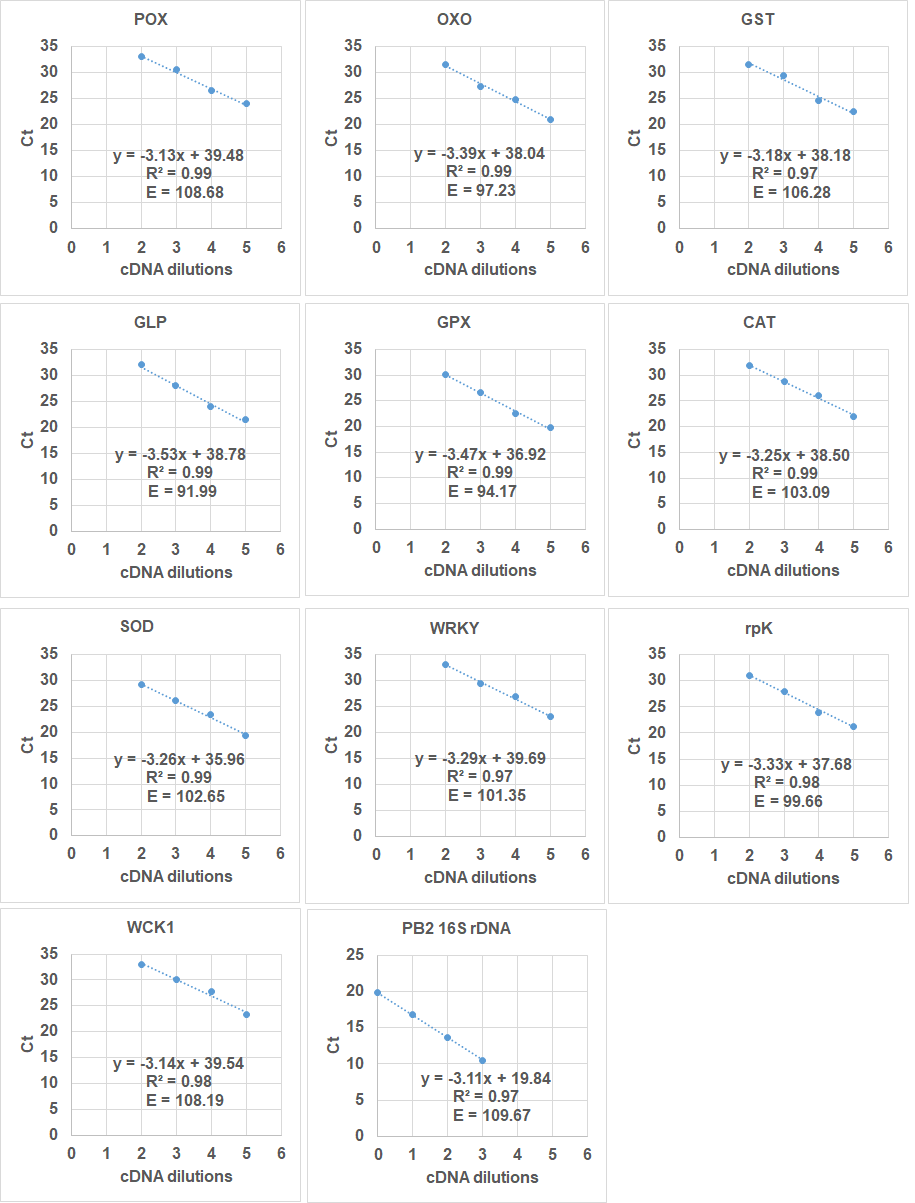

Supplement: FIGURE S5 — PCR’s amplification efficiency (E), for each primer pair used in the gene expression study and in the quantification of Paenibacillus strain B2, is deducted from the slopes (S) of the standard curves based on E = 100∗(10-1/s-1). Peroxidase (POX), oxalate oxidase (OXO), glutathione-s-transferase (GST), germin-like-protein (GLP), glutathione peroxidase (GPX), catalase (CAT), superoxide dismutase (SOD), related protein kinase (rpK), WRKY1 transcription factor (WRKY), MAP kinase (WCK1), and Paenibacillus strain B2 16S ribosomal DNA (PB2 16S rDNA). [file Image_5.TIF]

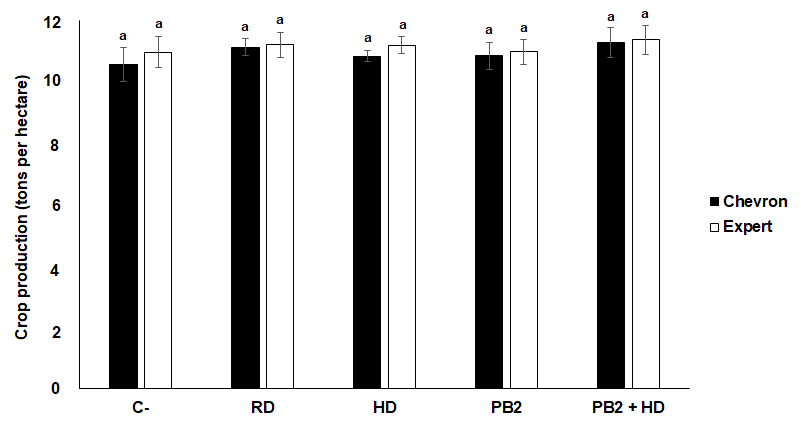

Supplement: FIGURE S6 — Field trials grain yield production the two cultivars, Expert and Chevron, as a response to wheat grains’ inoculation with Paenibacillus sp. strain B2, Cherokee® fungicide application, in recommended dose (RD) and half the recommended dose (HD), an association between PB2 and Cherokee® in HD (PB2+HD), and in PB2-non-inoculated and fungicide-non-treated controls (C-). The values shown are the means of one biological replicates and five technical replicates. Bars indicate means ± standard deviations. Different lower-case letters indicate significant differences between treatments, according to ANOVA followed by Tukey’s post hoc test (α = 0.05). [file Image_6.TIF]
