# Supplementary material for: The Effect of Plant Genotype, Growth Stage, and Mycosphaerella graminicola Strains on the Efficiency and Durability of Wheat-Induced Resistance by Paenibacillus sp. Strain B2
Source: Front Plant Sci. 2019 May 9;10:587. doi: 10.3389/fpls.2019.00587 (PMC6521617; doi:10.3389/fpls.2019.00587)
Supplement: TABLE S1 — Gene expression ratio of some wheat-defense-related genes encoding proteins from different classes, estimated by real-time PCR. [file Table_1.docx]

Supplementary Table 1. Gene expression ratio of some wheat-defense-related genes encoding proteins from different classes, estimated by real-time PCR.

| Gene | C/A | Alixan | | | | | | | | | | | | |
| --- | --- | --- | --- | --- | --- | --- | --- | --- | --- | --- | --- | --- | --- | --- |
|  |  | T0 | *M. graminicola* strain TO256 | | | | | | *M. graminicola* strain IPO323 | | | | | |
|  |  |  | 1 dai | | | 3 dai | | | 1 dai | | | 3 dai | | |
|  |  | PB2 | PB2 | MG | PB2/MG | PB2 | MG | PB2/MG | PB2 | MG | PB2/MG | PB2 | MG | PB2/MG |
| PR1 | 2.40 ± 1.03 | 2.59 ± 0.39 | 1.94 ± 1.37 | 0.94 ± 0.33 | 3.54 ± 1.76 | 1.81 ± 0.47 | 1.81 ± 0.65 | 1.36 ± 0.16 | 1.94 ± 1.37 | 6.83 ± 0.21 | 8.76 ± 4.33 | 1.81 ± 0.47 | 0.79 ± 0.54 | 0.21 ± 0.17 |
| CHIT | 2.87 ± 0.96 | 2.01 ± 0.68 | 0.71 ± 0.51 | 0.67 ± 0.34 | 1.20 ± 0.13 | 1.16 ± 0.25 | 3.18 ± 1.03 | 0.48 ± 0.51 | 0.71 ± 0.51 | 1.39 ± 0.58 | 2.19 ± 0.16 | 1.16 ± 0.25 | 0.81 ± 0.46 | 0.60 ± 0.10 |
| GLU | 0.83 ± 0.32 | 0.53 ± 0.16 | 0.73 ± 0.43 | 1.47 ± 0.17 | 2.44 ± 1.08 | 2.38 ± 0.72 | 0.84 ± 0.42 | 1.71 ± 0.65 | 0.73 ± 0.43 | 0.5 ± 0.38 | 0.35 ± 0.06 | 2.38 ± 0.72 | 0.17 ± 0.06 | 0.14 ± 0.09 |
| TLP | 0.98 ± 0.56 | 1.5 ± 0.18 | 0.88 ± 0.39 | 2.90 ± 0.65 | 1.42 ± 0.55 | 1.61 ± 0.38 | 0.08 ± 0.02 | 4.92 ± 1.31 | 0.88 ± 0.39 | 0.47 ± 0.34 | 1.84 ± 0.97 | 1.61 ± 0.38 | 0.71 ± 0.31 | 0.31 ± 0.20 |
| LIP | 1.13 ± 0.45 | 1.76 ± 0.48 | 1.01 ± 0.23 | 0.79 ± 0.25 | 0.82 ± 0.09 | 0.46 ± 0.37 | 0.49 ± 0.01 | 0.51 ± 0.13 | 1.01 ± 0.23 | 5.32 ± 3.03 | 0.15 ± 0.08 | 0.46 ± 0.37 | 4.11 ± 1.17 | 3.38 ± 0.20 |
| LOX | 3.55 ± 0.70 | 11.55 ± 3.84 | 0.19 ± 0.12 | 0.56 ± 0.27 | 1.60 ± 0.26 | 5.33 ± 1.07 | 2.71 ± 1.17 | 6.67 ± 1.94 | 0.19 ± 0.12 | 3.33 ± 0.23 | 0.79 ± 0.27 | 5.33 ± 1.07 | 0.89 ± 0.04 | 2.75 ± 0.75 |
| AOS | 0.60 ± 0.20 | 0.94 ± 0.24 | 2.37 ± 0.18 | 1.74 ± 1.09 | 2.14 ± 0.62 | 1.07 ± 0.46 | 0.88 ± 0.30 | 1.00 ± 0.29 | 2.37 ± 0.18 | 0.35 ± 0.11 | 0.24 ± 0.17 | 1.07 ± 0.46 | 1.04 ± 0.19 | 1.24 ± 0.43 |
| PAL | 6.80 ± 1.91 | 5.06 ± 1.41 | 1.17 ± 0.67 | 4.07 ± 0.75 | 2.48 ± 0.98 | 0.64 ± 0.22 | 0.76 ± 0.23 | 0.55 ± 0.20 | 1.17 ± 0.67 | 0.7 ± 0.46 | 0.54 ± 0.50 | 0.64 ± 0.22 | 0.91 ± 0.20 | 0.91 ± 0.16 |
| CHS | 2.59 ± 0.55 | 2.84 ± 1.11 | 0.73 ± 0.27 | 0.74 ± 0.24 | 0.83 ± 0.09 | 0.65 ± 0.11 | 1.09 ± 0.40 | 0.67 ± 0.26 | 0.73 ± 0.27 | 0.37 ± 0.13 | 1.13 ± 1.30 | 0.65 ± 0.11 | 1.38 ± 0.16 | 1.59 ± 0.40 |
| FLAV | 2.09 ± 1.08 | 1.87 ± 0.59 | 0.84 ± 0.42 | 1.67 ± 0.30 | 1.88 ± 0.44 | 1.73 ± 0.38 | 1.84 ± 0.51 | 2.41 ± 0.74 | 0.84 ± 0.42 | 3.87 ± 2.31 | 1.43 ± 0.94 | 1.73 ± 0.38 | 0.78 ± 0.54 | 0.32 ± 0.14 |
| POX | 1.09 ± 0.31 | 0.73 ± 0.30 | 0.45 ± 0.24 | 9.97 ± 3.99 | 1.01 ± 0.01 | 0.06 ± 0.05 | 0.02 ± 0.01 | 0.15 ± 0.18 | 0.45 ± 0.24 | 0.98 ± 0.76 | 2.54 ± 1.34 | 0.06 ± 0.05 | 1.47 ± 0.32 | 1.53 ± 0.36 |
| OXO | 3.31 ± 0.45 | 1.91 ± 0.55 | 1.32 ± 0.45 | 3.07 ± 0.88 | 3.78 ± 2.04 | 1.00 ± 0.71 | 1.02 ± 0.39 | 0.43 ± 0.09 | 1.32 ± 0.45 | 8.38 ± 4.77 | 4.27 ± 2.04 | 1.00 ± 0.71 | 0.98 ± 0.24 | 1.14 ± 0.28 |
| GST | 3.72 ± 0.58 | 2.3 ± 0.45 | 1.21 ± 0.32 | 1.27 ± 0.22 | 1.34 ± 0.07 | 1.42 ± 0.44 | 1.11 ± 0.09 | 0.38 ± 0.20 | 1.21 ± 0.32 | 1.31 ± 0.14 | 2.18 ± 1.17 | 1.42 ± 0.44 | 1.65 ± 0.42 | 1.98 ± 0.12 |
| GLP | 1.35 ± 0.26 | 0.98 ± 0.13 | 0.75 ± 0.25 | 1.68 ± 0.19 | 0.90 ± 0.42 | 1.05 ± 0.47 | 1.07 ± 0.71 | 0.98 ± 0.09 | 0.75 ± 0.25 | 0.69 ± 0.43 | 0.50 ± 0.20 | 1.05 ± 0.47 | 1.60 ± 0.66 | 1.61 ± 0.10 |
| GPX | 12.35 ± 1.36 | 35.1 ± 13.5 | 57.74 ± 11.10 | 3.25 ± 0.04 | 61.78 ± 14.69 | 1.09 ± 0.18 | 1.57 ± 0.26 | 1.30 ± 0.12 | 57.74 ± 11.10 | 0.47 ± 0.34 | 0.55 ± 0.46 | 1.09 ± 0.18 | 0.81 ± 0.09 | 0.74 ± 0.18 |
| CAT | 1.18 ± 0.31 | 0.89 ± 0.14 | 0.67 ± 0.21 | 0.95 ± 0.21 | 0.67 ± 0.08 | 0.78 ± 0.61 | 1.07 ± 0.55 | 0.46 ± 0.31 | 0.67 ± 0.21 | 0.69 ± 0.29 | 0.49 ± 0.34 | 0.78 ± 0.61 | 0.74 ± 0.04 | 0.73 ± 0.20 |
| SOD | 0.89 ± 0.09 | 1.12 ± 0.36 | 0.83 ± 0.39 | 1.02 ± 0.25 | 0.78 ± 0.18 | 1.00 ± 0.10 | 1.12 ± 0.06 | 1.00 ± 0.14 | 0.83 ± 0.39 | 0.31 ± 0.38 | 0.66 ± 0.60 | 1.00 ± 0.10 | 1.46 ± 0.29 | 1.16 ± 0.20 |
| rpK | 0.99 ± 0.11 | 0.78 ± 0.29 | 0.68 ± 0.27 | 0.68 ± 0.18 | 0.66 ± 0.21 | 0.17 ± 0.09 | 0.67 ± 0.61 | 0.20 ± 0.12 | 0.68 ± 0.27 | 0.59 ± 0.26 | 0.94 ± 0.09 | 0.17 ± 0.09 | 1.35 ± 0.37 | 1.06 ± 0.27 |
| WRKY | 1.20 ± 0.38 | 1.06 ± 0.27 | 0.79 ± 0.40 | 1.12 ± 0.06 | 0.58 ± 0.11 | 1.66 ± 0.76 | 0.55 ± 0.54 | 1.53 ± 0.41 | 0.79 ± 0.40 | 0.33 ± 0.18 | 0.63 ± 0.27 | 1.66 ± 0.76 | 0.97 ± 0.05 | 0.88 ± 0.22 |
| WCK1 | 1.53 ± 0.18 | 1.73 ± 0.06 | 0.77 ± 0.24 | 1.08 ± 0.31 | 0.60 ± 0.14 | 0.49 ± 0.15 | 1.02 ± 0.15 | 0.70 ± 0.46 | 0.77 ± 0.24 | 0.5 ± 0.26 | 0.21 ± 0.24 | 0.49 ± 0.15 | 0.85 ± 0.07 | 1.02 ± 0.08 |

Supplementary Table 1 Continued.

| Gene | Cellule | | | | | | | | | | | | |
| --- | --- | --- | --- | --- | --- | --- | --- | --- | --- | --- | --- | --- | --- |
|  | T0 | *M. graminicola* strain TO256 | | | | | | *M. graminicola* strain IPO323 | | | | | |
|  |  | 1 dai | | | 3 dai | | | 1 dai | | | 3 dai | | |
|  | PB2 | PB2 | MG | PB2/MG | PB2 | MG | PB2/MG | PB2 | MG | PB2/MG | PB2 | MG | PB2/MG |
| PR1 | 0.42 ± 0.25 | 1.45 ± 0.45 | 0.44 ± 0.23 | 0.85 ± 0.45 | 0.32 ± 0.11 | 1.17 ± 0.85 | 2.93 ± 1.15 | 1.45 ± 0.45 | 1.48 ± 0.68 | 5.11 ± 0.84 | 0.32 ± 0.11 | 0.8 ± 0.42 | 4.65 ± 1.50 |
| CHIT | 0.60 ± 0.07 | 7.45 ± 0.33 | 2.02 ± 0.62 | 1.10 ± 0.18 | 0.69 ± 0.42 | 8.06 ± 2.16 | 5.06 ± 0.93 | 7.45 ± 0.33 | 3.25 ± 0.13 | 0.96 ± 0.63 | 0.69 ± 0.42 | 1.44 ± 0.33 | 2.01 ± 0.26 |
| GLU | 0.35 ± 0.23 | 1.79 ± 0.71 | 1.50 ± 1.05 | 0.53 ± 0.39 | 0.57 ± 0.40 | 3.36 ± 1.39 | 2.60 ± 1.13 | 1.79 ± 0.71 | 5.24 ± 1.83 | 4.95 ± 1.99 | 0.57 ± 0.40 | 10.37 ± 5.93 | 22.94 ± 6.40 |
| TLP | 0.67 ± 0.47 | 0.09 ± 0.02 | 1.69 ± 0.34 | 0.73 ± 0.39 | 0.64 ± 0.35 | 0.65 ± 0.45 | 0.66 ± 0.66 | 0.09 ± 0.02 | 0.85 ± 0.26 | 0.82 ± 0.60 | 0.64 ± 0.35 | 1.04 ± 0.19 | 3.09 ± 1.56 |
| LIP | 1.54 ± 0.07 | 0.18 ± 0.03 | 2.09 ± 0.55 | 0.75 ± 0.48 | 0.91 ± 0.93 | 0.73 ± 0.07 | 0.34 ± 0.08 | 0.18 ± 0.03 | 2.51 ± 0.54 | 1.32 ± 0.08 | 0.91 ± 0.93 | 4.93 ± 0.73 | 5.15 ± 2.11 |
| LOX | 1.00 ± 0.26 | 3.85 ± 1.06 | 0.92 ± 0.22 | 0.45 ± 0.16 | 1.55 ± 1.3 | 5.55 ± 1.20 | 7.12 ± 1.96 | 3.85 ± 1.06 | 0.96 ± 0.67 | 0.38 ± 0.46 | 1.55 ± 1.3 | 2.41 ± 0.36 | 1.06 ± 0.78 |
| AOS | 0.46 ± 0.08 | 2.05 ± 0.43 | 1.15 ± 0.35 | 0.24 ± 0.10 | 0.39 ± 0.41 | 1.88 ± 0.05 | 1.83 ± 0.66 | 2.05 ± 0.43 | 2.43 ± 0.38 | 0.87 ± 0.57 | 0.39 ± 0.41 | 17.33 ± 8.98 | 13.71 ± 1.15 |
| PAL | 1.20 ± 0.45 | 1.08 ± 0.25 | 1.77 ± 0.19 | 0.49 ± 0.12 | 0.70 ± 0.56 | 1.50 ± 0.40 | 0.83 ± 0.17 | 1.08 ± 0.25 | 2.28 ± 0.44 | 2.29 ± 1.12 | 0.70 ± 0.56 | 17.21 ± 3.44 | 29.54 ± 2.51 |
| CHS | 1.44 ± 0.21 | 1.02 ± 0.10 | 1.09 ± 0.42 | 1.36 ± 0.62 | 1.35 ± 0.73 | 0.95 ± 0.49 | 0.96 ± 0.22 | 1.02 ± 0.10 | 1.13 ± 0.18 | 0.72 ± 0.44 | 1.35 ± 0.73 | 1.21 ± 0.45 | 1.38 ± 0.04 |
| FLAV | 0.36 ± 0.40 | 0.13 ± 0.02 | 2.65 ± 0.22 | 0.95 ± 0.58 | 0.65 ± 0.46 | 1.15 ± 0.02 | 0.80 ± 0.54 | 0.13 ± 0.02 | 1.38 ± 0.37 | 5.08 ± 0.31 | 0.65 ± 0.46 | 14.89 ± 2.50 | 48.29 ± 18.69 |
| POX | 0.93 ± 0.42 | 0.25 ± 0.18 | 3.84 ± 1.28 | 0.60 ± 0.55 | 0.40 ± 0.32 | 0.14 ± 0.11 | 0.44 ± 0.59 | 0.25 ± 0.18 | 18.84 ± 6.57 | 7.35 ± 4.42 | 0.40 ± 0.32 | 5.99 ± 2.90 | 11.4 ± 0.62 |
| OXO | 2.07 ± 0.44 | 0.63 ± 0.18 | 1.51 ± 0.18 | 1.58 ± 0.53 | 0.72 ± 0.19 | 0.58 ± 0.47 | 0.48 ± 0.08 | 0.63 ± 0.18 | 4.32 ± 0.98 | 3.35 ± 1.05 | 0.72 ± 0.19 | 1.55 ± 0.28 | 2.15 ± 0.56 |
| GST | 0.53 ± 0.04 | 1.38 ± 0.91 | 1.78 ± 0.26 | 1.71 ± 0.56 | 1.73 ± 0.11 | 3.16 ± 1.16 | 0.79 ± 0.33 | 1.38 ± 0.91 | 1.96 ± 0.44 | 1.79 ± 0.20 | 1.73 ± 0.11 | 2.27 ± 0.75 | 2.40 ± 0.30 |
| GLP | 1.01 ± 0.14 | 0.18 ± 0.05 | 2.15 ± 1.13 | 0.58 ± 0.09 | 0.57 ± 0.41 | 1.07 ± 0.19 | 0.90 ± 0.25 | 0.18 ± 0.05 | 5.26 ± 0.81 | 4.01 ± 0.58 | 0.57 ± 0.41 | 2.91 ± 0.47 | 3.60 ± 0.59 |
| GPX | 3.76 ± 1.24 | 0.32 ± 0.18 | 1.04 ± 0.40 | 0.43 ± 0.39 | 0.62 ± 0.51 | 0.23 ± 0.14 | 0.77 ± 0.53 | 0.32 ± 0.18 | 0.63 ± 0.36 | 0.38 ± 0.37 | 0.62 ± 0.51 | 1.55 ± 0.08 | 1.93 ± 0.19 |
| CAT | 0.67 ± 0.08 | 0.42 ± 0.60 | 1.75 ± 0.46 | 1.15 ± 0.04 | 0.86 ± 0.51 | 0.48 ± 0.32 | 0.49 ± 0.34 | 0.42 ± 0.60 | 2.22 ± 0.05 | 3.25 ± 0.62 | 0.86 ± 0.51 | 1.31 ± 0.02 | 1.64 ± 0.36 |
| SOD | 0.88 ± 0.27 | 1.05 ± 0.06 | 1.04 ± 0.28 | 0.47 ± 0.24 | 0.70 ± 0.61 | 1.08 ± 0.05 | 1.00 ± 0.23 | 1.05 ± 0.06 | 0.19 ± 0.12 | 0.21 ± 0.08 | 0.70 ± 0.61 | 1.07 ± 0.14 | 0.83 ± 0.17 |
| rpK | 0.67 ± 0.20 | 1.17 ± 0.03 | 0.85 ± 0.28 | 0.38 ± 0.17 | 0.50 ± 0.39 | 1.29 ± 0.66 | 9.24 ± 3.12 | 1.17 ± 0.03 | 0.20 ± 0.26 | 2.78 ± 1.06 | 0.50 ± 0.39 | 0.97 ± 0.16 | 1.16 ± 0.02 |
| WRKY | 0.90 ± 0.18 | 0.44 ± 0.22 | 2.94 ± 0.74 | 0.60 ± 0.30 | 0.61 ± 0.44 | 0.27 ± 0.23 | 0.15 ± 0.08 | 0.44 ± 0.22 | 3.05 ± 1.04 | 7.73 ± 0.25 | 0.61 ± 0.44 | 2.52 ± 0.54 | 3.22 ± 0.28 |
| WCK1 | 0.95 ± 0.24 | 0.21 ± 0.04 | 3.11 ± 0.88 | 0.74 ± 0.25 | 0.89 ± 0.74 | 0.77 ± 0.02 | 0.65 ± 0.21 | 0.21 ± 0.04 | 1.39 ± 1.36 | 0.57 ± 0.39 | 0.89 ± 0.74 | 7.56 ± 0.95 | 10.27 ± 2.40 |

The values shown are the mean and standard deviation of three biological replicates and five technical replicates. Relative gene expressions were determined in the leaves of the moderately resistant cultivar Cellule compared to the susceptible cultivar Alixan (C/A) or as a response to root inoculation with *Paenibacillus* sp. strain B2 (PB2); leaves infected with *M. graminicola* (MG), or inoculated with PB2 and infected with MG (PB2/MG) were compared to control modalities without PB2 and MG. Gene expressions were studied at 3-leaf growth stage, at the moment of leaves’ infection with *M. graminicola* (T0), 1 and 3 days after inoculation (dai).
