# Supplementary material for: The Effect of Plant Genotype, Growth Stage, and Mycosphaerella graminicola Strains on the Efficiency and Durability of Wheat-Induced Resistance by Paenibacillus sp. Strain B2
Source: Front Plant Sci. 2019 May 9;10:587. doi: 10.3389/fpls.2019.00587 (PMC6521617; doi:10.3389/fpls.2019.00587)
Supplement: TABLE S2 — Gene expression ratio in the susceptible cultivar Alixan as a response to Paenibacillus B2 (PB2), M. graminicola strain IPO323 (MG) and Paenibacillus B2 and M. graminicola strain IPO323 (PB2/MG), at the time of infection with IPO323 (T0), 6, 12, 24, and 48 h after inoculation (hai), 3, 5, 9, and 11 days after inoculation (dai). [file Table_2.DOCX]

Supplementary Table 2. Gene expression ratio in the susceptible cultivar Alixan as a response to *Paenibacillus* B2 (PB2), *M. graminicola* strain IPO323 (MG) and *Paenibacillus* B2 and *M. graminicola* strain IPO323 (PB2/MG), at the time of infection with IPO323 (T0), 6, 12, 24, 48 hours after inoculation (hai), 3, 5, 9, and 11 days after inoculation (dai).

| Gene | PB2 | | | | | | | | |
| --- | --- | --- | --- | --- | --- | --- | --- | --- | --- |
|  | T0 | 6 hai | 12 hai | 24 hai | 48 hai | 3 dai | 5 dai | 9 dai | 11 dai |
| PR1 | 2.59 ± 0.39 | 0.57 ± 0.02 | 3.73 ± 1.27 | 1.29 ± 0.42 | 0.99 ± 0.31 | 0.64 ± 0.57 | 0.50 ± 0.12 | 2.16 ± 0.97 | 0.90 ± 0.66 |
| CHIT | 2.01 ± 0.68 | 0.61 ± 0.60 | 1.08 ± 0.26 | 0.90 ± 0.07 | 1.23 ± 0.27 | 0.91 ± 0.40 | 0.63 ± 0.12 | 0.55 ± 0.14 | 0.91 ± 0.42 |
| GLU | 0.53 ± 0.16 | 0.85 ± 0.49 | 0.51 ± 0.10 | 0.21 ± 0.21 | 0.98 ± 0.19 | 1.15 ± 0.25 | 0.38 ± 0.31 | 0.49 ± 0.58 | 5.24 ± 1.60 |
| TLP | 1.5 ± 0.18 | 0.46 ± 0.26 | 2.00 ± 0.74 | 0.72 ± 0.34 | 0.86 ± 0.46 | 1.25 ± 0.20 | 0.47 ± 0.20 | 1.03 ± 0.33 | 1.32 ± 0.48 |
| LIP | 1.76 ± 0.48 | 0.60 ± 0.64 | 0.82 ± 0.30 | 1.39 ± 0.29 | 1.34 ± 0.48 | 3.20 ± 0.63 | 1.36 ± 0.20 | 0.71 ± 0.19 | 1.53 ± 0.35 |
| LOX | 11.55 ± 3.84 | 1.14 ± 1.28 | 0.83 ± 0.32 | 0.51 ± 0.33 | 2.61 ± 1.18 | 1.55 ± 0.54 | 0.28 ± 0.19 | 0.58 ± 0.13 | 5.18 ± 2.47 |
| AOS | 0.94 ± 0.24 | 0.34 ± 0.26 | 0.69 ± 0.21 | 0.80 ± 0.12 | 1.52 ± 0.74 | 0.97 ± 0.20 | 2.22 ± 0.29 | 0.60 ± 0.22 | 1.34 ± 0.30 |
| PAL | 5.06 ± 1.41 | 0.53 ± 0.17 | 0.95 ± 0.65 | 0.57 ± 0.13 | 2.36 ± 0.74 | 0.90 ± 0.28 | 1.09 ± 0.17 | 0.18 ± 0.07 | 1.02 ± .020 |
| CHS | 2.84 ± 1.11 | 1.92 ± 1.65 | 0.62 ± 0.12 | 0.99 ± 0.14 | 0.76 ± 0.37 | 1.01 ± 0.08 | 0.88 ± 0.15 | 0.93 ± 0.19 | 1.13 ± 0.22 |
| FLAV | 1.87 ± 0.59 | 2.18 ± 1.36 | 2.23 ± 0.88 | 1.43 ± 1.05 | 0.75 ± 0.22 | 2.51 ± 1.05 | 0.24 ± 0.13 | 0.41 ± 0.23 | 1.14 ± 0.63 |
| POX | 0.73 ± 0.30 | 2.20 ± 1.43 | 0.46 ± 0.09 | 0.76 ± 0.29 | 0.66 ± 0.46 | 0.98 ± 0.57 | 0.79 ± 0.61 | 0.23 ± 0.05 | 2.57 ± 0.39 |
| OXO | 1.91 ± 0.55 | 0.30 ± 0.16 | 0.94 ± 0.23 | 0.96 ± 0.22 | 0.84 ± 0.12 | 1.51 ± 0.24 | 0.58 ± 0.11 | 0.76 ± 0.12 | 0.98 ± 0.43 |
| GST | 2.3 ± 0.45 | 1.80 ± 0.84 | 1.13 ± 0.24 | 1.02 ± 0.10 | 0.74 ± 0.35 | 2.22 ± 0.18 | 1.11 ± 0.20 | 0.76 ± 0.10 | 0.94 ± 0.15 |
| GLP | 0.98 ± 0.13 | 0.62 ± 0.44 | 0.75 ± 0.15 | 0.93 ± 0.07 | 1.08 ± 0.24 | 2.07 ± 0.11 | 0.71 ± 0.21 | 0.63 ± 0.17 | 1.04 ± 0.16 |
| GPX | 35.1 ± 13.5 | 0.23 ± 0.31 | 0.68 ± 0.13 | 0.85 ± 0.14 | 1.00 ± 0.20 | 0.77 ± 0.06 | 0.54 ± 0.14 | 0.25 ± 0.43 | 1.01 ± 0.14 |
| CAT | 0.89 ± 0.14 | 0.76 ± 0.40 | 0.48 ± 0.09 | 0.30 ± 0.15 | 0.85 ± 0.04 | 0.95 ± 0.24 | 0.64 ± 0.11 | 1.01 ± 0.16 | 1.15 ± 0.09 |
| SOD | 1.12 ± 0.36 | 0.23 ± 0.31 | 0.68 ± 0.10 | 1.27 ± 0.17 | 0.96 ± 0.17 | 1.22 ± 0.12 | 0.72 ± 0.21 | 0.96 ± 0.28 | 0.89 ± 0.11 |
| rpK | 0.78 ± 0.29 | 0.76 ± 0.40 | 0.36 ± 0.16 | 1.15 ± 0.30 | 1.00 ± 0.14 | 1.18 ± 0.15 | 0.65 ± 0.04 | 0.79 ± 0.29 | 1.03 ± 0.30 |
| WRKY1 | 1.06 ± 0.27 | 0.41 ± 0.23 | 0.60 ± 0.18 | 1.20 ± 0.23 | 1.26 ± 0.46 | 1.31 ± 0.19 | 0.67 ± 0.11 | 0.65 ± 0.20 | 0.70 ± 0.13 |
| WCK1 | 1.73 ± 0.06 | 0.53 ± 0.18 | 0.55 ± 0.18 | 0.78 ± 0.15 | 0.96 ± 0.53 | 0.97 ± 0.23 | 0.66 ± 0.09 | 0.48 ± 0.23 | 0.91 ± 0.11 |

Supplementary Table 2 continued.

| Gene | MG | | | | | | | |
| --- | --- | --- | --- | --- | --- | --- | --- | --- |
|  | 6 hai | 12 hai | 24 hai | 48 hai | 3 dai | 5 dai | 9 dai | 11 dai |
| PR1 | 2.24 ± 0.90 | 4.25 ± 0.79 | 10.24 ± 4.82 | 1.57 ± 0.82 | 0.79 ± 0.54 | 1.67 ± 0.73 | 4.36 ± 2.46 | 1.70 ± 0.16 |
| CHIT | 0.8 ± 0.20 | 1.03 ± 0.24 | 1.39 ± 0.58 | 1.21 ± 0.35 | 0.81 ± 0.46 | 0.67 ± 0.16 | 0.31 ± 0.06 | 1.13 ± 0.41 |
| GLU | 1.41 ± 1.3 | 4.98 ± 1.66 | 0.50 ± 0.38 | 3.72 ± 0.97 | 0.17 ± 0.06 | 0.25 ± 0.08 | 0.45 ± 0.28 | 1.71 ± 0.31 |
| TLP | 6.88 ± 1.65 | 4.17 ± 1.52 | 0.47 ± 0.34 | 2.12 ± 0.62 | 0.71 ± 0.31 | 0.73 ± 0.23 | 1.36 ± 0.57 | 3.01 ± 0.63 |
| LIP | 0.56 ± 0.08 | 0.38 ± 0.17 | 5.32 ± 3.03 | 1.18 ± 0.46 | 4.11 ± 1.17 | 2.02 ± 0.06 | 0.97 ± 0.14 | 3.09 ± 1.13 |
| LOX | 0.82 ± 1.14 | 1.02 ± 0.67 | 3.33 ± 0.23 | 2.42 ± 1.80 | 0.89 ± 0.04 | 0.50 ± 0.36 | 0.34 ± 0.17 | 1.10 ± 0.32 |
| AOS | 0.33 ± 0.36 | 1.20 ± 0.48 | 0.35 ± 0.11 | 2.05 ± 0.90 | 1.04 ± 0.19 | 2.10 ± 0.70 | 0.26 ± 0.11 | 1.13 ± 0.31 |
| PAL | 3.96 ± 3.23 | 0.61 ± 0.21 | 0.70 ± 0.46 | 3.65 ± 2.25 | 0.91 ± 0.20 | 1.11 ± 0.34 | 0.09 ± 0.04 | 0.63 ± 0.27 |
| CHS | 7.85 ± 2.00 | 0.37 ± 0.22 | 0.37 ± 0.13 | 0.80 ± 0.27 | 1.38 ± 0.16 | 0.81 ± 0.16 | 0.81 ± 0.20 | 0.87 ± 0.19 |
| FLAV | 5.28 ± 1.25 | 8.18 ± 2.26 | 22.85 ± 9.93 | 1.26 ± 0.42 | 0.78 ± 0.54 | 0.64 ± 0.29 | 0.68 ± 0.23 | 0.75 ± 0.15 |
| POX | 6.29 ± 2.96 | 3.45 ± 0.58 | 0.76 ± 00.12 | 3.00 ± 0.55 | 1.47 ± 0.32 | 0.86 ± 0.11 | 0.56 ± 0.17 | 2.73 ± 0.78 |
| OXO | 0.42 ± 0.03 | 1.79 ± 0.92 | 8.38 ± 2.78 | 1.16 ± 0.25 | 0.98 ± 0.24 | 0.90 ± 0.18 | 0.71 ± 0.20 | 0.95 ± 0.34 |
| GST | 0.94 ± 0.09 | 1.15 ± 0.46 | 1.31 ± 0.14 | 1.02 ± 0.02 | 1.65 ± 0.42 | 1.03 ± 0.23 | 0.83 ± 0.17 | 1.00 ± 0.26 |
| GLP | 1.62 ± 0.32 | 1.47 ± 0.70 | 0.69 ± 0.43 | 1.07 ± 0.33 | 1.60 ± 0.66 | 0.75 ± 0.20 | 0.63 ± 0.31 | 1.66 ± 0.67 |
| GPX | 0.83 ± 0.29 | 0.71 ± 0.35 | 0.47 ± 0.34 | 1.02 ± 0.21 | 0.81 ± 0.09 | 0.78 ± 0.17 | 0.54 ± 0.14 | 1.38 ± 0.47 |
| CAT | 0.68 ± 0.03 | 0.52 ± 0.27 | 0.69 ± 0.29 | 0.99 ± 0.09 | 0.74 ± 0.04 | 0.63 ± 0.14 | 0.81 ± 0.24 | 1.10 ± 0.16 |
| SOD | 0.65 ± 0.46 | 0.62 ± 0.14 | 0.31 ± 0.38 | 0.94 ± 0.31 | 1.46 ± 0.29 | 0.98 ± 0.33 | 1.05 ± 0.40 | 1.07 ± 0.29 |
| rpK | 1.00 ± 0.57 | 0.31 ± 0.13 | 0.59 ± 0.54 | 0.88 ± 0.33 | 1.35 ± 0.37 | 0.88 ± 0.20 | 0.73 ± 0.28 | 1.25 ± 0.39 |
| WRKY1 | 1.14 ± 0.26 | 0.26 ± 0.11 | 0.33 ± 0.18 | 0.98 ± 0.25 | 0.97 ± 0.05 | 0.83 ± 0.29 | 0.46 ± 0.13 | 1.05 ± 0.25 |
| WCK1 | 0.84 ± 0.09 | 0.42 ± 0.06 | 0.50 ± 0.26 | 0.83 ± 0.20 | 0.85 ± 0.07 | 0.96 ± 0.17 | 0.27 ± 0.12 | 1.31 ± 0.33 |

Supplementary Table 2 continued.

| Gene | PB2/MG | | | | | | | |
| --- | --- | --- | --- | --- | --- | --- | --- | --- |
|  | 6 hai | 12 hai | 24 hai | 48 hai | 3 dai | 5 dai | 9 dai | 11 dai |
| PR1 | 5.11 ± 0.21 | 5.55 ± 2.66 | 8.72 ± 4.33 | 2.38 ± 0.78 | 0.21 ± 0.17 | 2.09 ± 0.58 | 2.13 ± 0.62 | 0.72 ± 0.08 |
| CHIT | 7.05 ± 1.4 | 1.25 ± 0.51 | 2.19 ± 0.16 | 1.11 ± 0.34 | 0.60 ± 0.10 | 0.55 ± 0.03 | 0.45 ± 0.17 | 0.87 ± 0.29 |
| GLU | 10.26 ± 0.03 | 9.76 ± 2.06 | 0.35 ± 0.06 | 3.09 ± 1.59 | 0.14 ± 0.09 | 0.18 ± 0.08 | 0.68 ± 0.59 | 3.32 ± 1.91 |
| TLP | 12.40 ± 1.27 | 5.66 ± 1.92 | 1.40 ± 0.97 | 2.03 ± 0.97 | 0.31 ± 0.20 | 1.09 ± 0.23 | 2.20 ± 0.58 | 2.52 ± 1.40 |
| LIP | 0.44 ± 0.18 | 0.49 ± 0.13 | 1.52 ± 1.95 | 1.18 ± 0.65 | 3.38 ± 0.20 | 1.86 ± 0.15 | 0.99 ± 0.06 | 1.70 ± 0.32 |
| LOX | 2.28 ± 0.70 | 1.16 ± 0.60 | 0.79 ± 0.27 | 2.06 ± 0.37 | 2.75 ± 0.75 | 0.47 ± 0.18 | 0.55 ± 0.38 | 10.45 ± 6.68 |
| AOS | 0.62 ± 0.31 | 0.71 ± 0.23 | 0.24 ± 0.17 | 0.95 ± 0.13 | 1.24 ± 0.43 | 1.62 ± 0.46 | 0.67 ± 0.38 | 2.45 ± 0.35 |
| PAL | 1.24 ± 0.43 | 0.47 ± 0.11 | 0.54 ± 0.50 | 1.70 ± 0.40 | 0.91 ± 0.16 | 0.88 ± 0.30 | 0.30 ± 0.20 | 1.51 ± 0.22 |
| CHS | 0.87 ± 0.08 | 0.36 ± 0.16 | 1.13 ± 1.30 | 0.61 ± 0.14 | 1.36 ± 0.53 | 0.80 ± 0.24 | 0.97 ± 0.32 | 0.91 ± 0.13 |
| FLAV | 14.96 ± 2.27 | 10.86 ± 4.15 | 1.43 ± 0.94 | 0.74 ± 0.25 | 0.32 ± 0.14 | 0.60 ± 0.39 | 0.46 ± 0.21 | 1.88 ± 0.24 |
| POX | 4.01 ± 1.70 | 3.31 ± 0.46 | 8.04 ± 1.34 | 1.89 ± 0.67 | 1.53 ± 0.36 | 0.65 ± 0.07 | 0.57 ± 0.34 | 1.92 ± 1.01 |
| OXO | 1.20 ± 0.52 | 1.96 ± 0.72 | 4.27 ± 2.05 | 1.02 ± 0.21 | 1.14 ± 0.28 | 0.60 ± 0.18 | 1.09 ± 0.08 | 0.86 ± 0.17 |
| GST | 1.09 ± 0.27 | 0.80 ± 0.12 | 2.18 ± 1.17 | 0.85 ± 0.23 | 1.98 ± 0.12 | 1.04 ± 0.14 | 0.60 ± 0.05 | 0.86 ± 0.17 |
| GLP | 4.10 ± 0.34 | 1.29 ± 0.56 | 3.50 ± 0.40 | 0.99 ± 0.34 | 1.61 ± 0.10 | 0.64 ± 0.08 | 0.80 ± 0.22 | 1.10 ± 0.31 |
| GPX | 0.81 ± 0.20 | 0.86 ± 0.36 | 0.55 ± 0.46 | 0.71 ± 0.19 | 0.74 ± 0.18 | 0.75 ± 0.08 | 0.79 ± 0.17 | 0.98 ± 0.14 |
| CAT | 0.70 ± 0.33 | 0.45 ± 0.18 | 0.49 ± 0.34 | 0.84 ± 0.22 | 0.73 ± 0.20 | 0.74 ± 0.10 | 0.99 ± 0.20 | 1.15 ± 0.13 |
| SOD | 0.81 ± 0.20 | 0.38 ± 0.14 | 0.66 ± 0.60 | 0.72 ± 0.18 | 1.16 ± 0.31 | 0.76 ± 0.19 | 0.68 ± 0.09 | 0.78 ± 0.08 |
| rpK | 0.70 ± 0.22 | 0.31 ± 0.09 | 0.94 ± 0.09 | 0.80 ± 0.21 | 1.06 ± 0.27 | 0.78 ± 0.16 | 0.71 ± 0.04 | 1.01 ± 0.04 |
| WRKY1 | 0.87 ± 0.29 | 0.35 ± 0.17 | 0.63 ± 0.27 | 0.62 ± 0.12 | 0.88 ± 0.22 | 0.78 ± 0.15 | 0.77 ± 0.21 | 0.76 ± 0.10 |
| WCK1 | 1.13 ± 0.15 | 0.38 ± 0.11 | 0.21 ± 0.24 | 0.74 ± 0.27 | 1.02 ± 0.08 | 0.82 ± 0.12 | 0.57 ± 0.22 | 0.97 ± 0.15 |

The values shown are the mean and standard deviation of three biological replicates and five technical replicates.
