# Supplementary material for: The Effect of Plant Genotype, Growth Stage, and Mycosphaerella graminicola Strains on the Efficiency and Durability of Wheat-Induced Resistance by Paenibacillus sp. Strain B2
Source: Front Plant Sci. 2019 May 9;10:587. doi: 10.3389/fpls.2019.00587 (PMC6521617; doi:10.3389/fpls.2019.00587)
Supplement: TABLE S3 — Gene expression ratio in the moderate cultivar (Cellule) as a response to Paenibacillus B2 (PB2), M. graminicola strain IPO323 (MG) and Paenibacillus B2 and M. graminicola strain IPO323 (PB2/MG), at the time of infection with IPO323 (T0), 6, 12, 24, and 48 h after inoculation (hai), 3, 5, 9, and 11 days after inoculation (dai). [file Table_3.DOCX]

Supplementary Table 3. Gene expression ratio in the moderate cultivar (Cellule) as a response to *Paenibacillus* B2 (PB2), *M. graminicola* strain IPO323 (MG) and *Paenibacillus* B2 and *M. graminicola* strain IPO323 (PB2/MG), at the time of infection with IPO323 (T0), 6, 12, 24, 48 hours after inoculation (hai), 3, 5, 9, and 11 days after inoculation (dai).

| Gene | PB2 | | | | | | | | |
| --- | --- | --- | --- | --- | --- | --- | --- | --- | --- |
|  | T0 | 6 hai | 12 hai | 24 hai | 48 hai | 3 dai | 5 dai | 9 dai | 11 dai |
| PR1 | 0.42 ± 0.25 | 1.48 ± 0.78 | 1.15 ± 0.13 | 1.17 ± 0.98 | 1.67 ± 0.43 | 0.90 ± 0.65 | 0.32 ± 0.16 | 1.25 ± 0.67 | 1.59 ± 1.23 |
| CHIT | 0.60 ± 0.07 | 7.50 ± 0.86 | 0.43 ± 0.09 | 1.11 ± 0.30 | 1.10 ± 0.41 | 4.48 ± 1.03 | 0.45 ± 0.12 | 1.05 ± 0.29 | 1.12 ± 0.22 |
| GLU | 0.35 ± 0.23 | 2.06 ± 1.17 | 0.92 ± 0.62 | 1.73 ± 1.23 | 2.81 ± 1.74 | 1.60 ± 0.96 | 0.29 ± 0.24 | 1.81 ± 1.53 | 3.67 ± 2.24 |
| TLP | 0.67 ± 0.47 | 1.91 ± 1.48 | 1.36 ± 0.19 | 0.43 ± 0.18 | 2.86 ± 0.54 | 2.55 ± 0.30 | 0.56 ± 0.74 | 1.58 ± 0.74 | 2.05 ± 0.86 |
| LIP | 1.54 ± 0.07 | 0.59 ± 0.25 | 1.45 ± 0.22 | 3.08 ± 1.89 | 2.53 ± 0.24 | 2.26 ± 0.76 | 0.65 ± 0.12 | 1.13 ± 0.16 | 1.71 ± 0.67 |
| LOX | 1.00 ± 0.26 | 5.93 ± 5.09 | 1.02 ± 0.30 | 0.65 ± 0.17 | 2.76 ± 1.20 | 2.18 ± 1.70 | 0.16 ± 0.03 | 0.55 ± 0.40 | 1.39 ± 0.48 |
| AOS | 0.46 ± 0.08 | 2.72 ± 0.84 | 1.50 ± 0.41 | 1.00 ± 0.44 | 1.97 ± 0.56 | 1.34 ± 1.44 | 0.68 ± 0.28 | 1.07 ± 0.5 | 1.44 ± 0.54 |
| PAL | 1.20 ± 0.45 | 1.81 ± 0.70 | 1.04 ± 0.27 | 1.17 ± 0.93 | 1.22 ± 0.40 | 2.36 ± 0.12 | 0.59 ± 0.27 | 1.08 ± 0.65 | 1.44 ± 0.33 |
| CHS | 1.44 ± 0.21 | 15.94 ± 4.87 | 0.75 ± 0.25 | 0.83 ± 0.25 | 0.79 ± 0.26 | 1.33 ± 0.66 | 1.11 ± 0.34 | 0.97 ± 0.37 | 1.34 ± 0.20 |
| FLAV | 0.36 ± 0.40 | 1.09 ± 0.41 | 1.40 ± 0.58 | 1.81 ± 0.33 | 3.94 ± 1.13 | 0.54 ± 0.64 | 0.24 ± 0.16 | 1.34 ± 0.40 | 0.90 ± 0.78 |
| POX | 0.93 ± 0.42 | 1.19 ± 0.99 | 0.97 ± 0.68 | 4.18 ± 2.92 | 0.76 ± 0.23 | 2.03 ± 0.70 | 0.29 ± 0.19 | 0.82 ± 0.88 | 0.83 ± 0.28 |
| OXO | 2.07 ± 0.44 | 0.84 ± 0.75 | 1.03 ± 0.14 | 0.99 ± 0.43 | 1.91 ± 0.79 | 1.42 ± 0.09 | 0.67 ± 0.21 | 1.09 ± 0.47 | 0.82 ± 0.21 |
| GST | 0.53 ± 0.04 | 3.51 ± 0.20 | 0.94 ± 0.16 | 0.90 ± 0.13 | 2.26 ± 1.10 | 1.40 ± 0.12 | 0.73 ± 0.11 | 0.63 ± 0.20 | 0.88 ± 0.09 |
| GLP | 1.01 ± 0.14 | 0.15 ± 0.10 | 0.98 ± 0.11 | 2.29 ± 1.03 | 1.83 ± 0.68 | 1.62 ± 0.71 | 0.50 ± 0.16 | 1.04 ± 0.31 | 1.00 ± 0.27 |
| GPX | 3.76 ± 1.24 | 0.28 ± 0.16 | 0.53 ± 0.14 | 0.42 ± 0.27 | 0.89 ± 0.27 | 0.85 ± 0.45 | 0.50 ± 0.12 | 1.15 ± 0.79 | 0.83 ± 0.22 |
| CAT | 0.67 ± 0.08 | 0.59 ± 0.24 | 0.74 ± 0.18 | 3.03 ± 1.30 | 0.58 ± 0.29 | 0.35 ± 0.22 | 0.83 ± 0.28 | 4.85 ± 2.15 | 0.88 ± 0.30 |
| SOD | 0.88 ± 0.27 | 0.21 ± 0.15 | 1.46 ± 0.20 | 0.21 ± 0.24 | 1.11 ± 0.07 | 1.14 ± 0.09 | 0.68 ± 0.14 | 1.04 ± 0.14 | 1.13 ± 0.10 |
| rpK | 0.67 ± 0.20 | 0.59 ± 0.24 | 1.03 ± 0.19 | 5.48 ± 2.47 | 1.08 ± 0.01 | 0.93 ± 0.45 | 0.59 ± 0.15 | 1.26 ± 0.15 | 1.13 ± 0.18 |
| WRKY1 | 0.90 ± 0.18 | 0.05 ± 0.01 | 1.08 ± 0.31 | 3.72 ± 1.29 | 1.60 ± 0.59 | 1.65 ± 0.21 | 0.62 ± 0.38 | 1.09 ± 0.38 | 0.95 ± 0.17 |
| WCK1 | 0.95 ± 0.24 | 1.18 ± 0.48 | 0.92 ± 0.22 | 0.36 ± 0.28 | 1.73 ± 0.17 | 0.58 ± 0.16 | 0.53 ± 0.31 | 0.62 ± 0.31 | 0.80 ± 0.24 |

Supplementary Table 3 continued.

| Gene | MG | | | | | | | |
| --- | --- | --- | --- | --- | --- | --- | --- | --- |
|  | 6h | 12h | 24h | 48h | 3j | 5j | 9j | 11j |
| PR1 | 2.21 ± 0.24 | 3.33 ± 2.67 | 1.48 ± 0.68 | 5.12 ± 2.22 | 0.80 ± 0.42 | 0.56 ± 0.16 | 1.86 ± 0.75 | 7.10 ± 2.37 |
| CHIT | 1.23 ± 1.30 | 0.98 ± 0.36 | 3.25 ± 0.13 | 3.01 ± 1.76 | 1.44 ± 0.33 | 0.47 ± 0.09 | 1.06 ± 0.45 | 2.36 ± 0.24 |
| GLU | 3.83 ± 1.30 | 3.93 ± 1.35 | 5.24 ± 1.83 | 0.71 ± 0.06 | 10.37 ± 5.93 | 0.16 ± 0.08 | 0.72 ± 0.43 | 8.38 ± 1.98 |
| TLP | 8.33 ± 2.89 | 7.05 ± 1.12 | 0.85 ± 0.28 | 6.58 ± 4.47 | 1.04 ± 0.19 | 0.63 ± 0.32 | 0.80 ± 0.09 | 9.50 ± 1.71 |
| LIP | 0.37 ± 0.19 | 0.93 ± 0.34 | 2.51 ± 0.54 | 1.77 ± 0.99 | 4.93 ± 0.73 | 0.77 ± 0.43 | 0.70 ± 0.34 | 1.49 ± 1.01 |
| LOX | 0.57 ± 0.08 | 2.58 ± 0.50 | 0.96 ± 0.67 | 2.05 ± 0.89 | 2.41 ± 0.36 | 0.80 ± 0.41 | 0.36 ± 0.17 | 6.42 ± 0.17 |
| AOS | 1.32 ± 0.99 | 1.94 ± 0.10 | 2.43 ± 0.38 | 1.08 ± 0.18 | 17.33 ± 8.98 | 0.62 ± 0.31 | 0.55 ± 0.27 | 2.04 ± 0.28 |
| PAL | 1.48 ± 0.89 | 1.63 ± 0.49 | 2.28 ± 0.44 | 1.69 ± 0.48 | 17.21 ± 3.44 | 0.47 ± 0.25 | 0.68 ± 0.22 | 1.47 ± 0.18 |
| CHS | 7.00 ± 5.44 | 0.99 ± 0.38 | 1.13 ± 0.18 | 1.98 ± 0.41 | 1.21 ± 0.45 | 0.71 ± 0.21 | 0.68 ± 0.27 | 1.00 ± 0.29 |
| FLAV | 6.07 ± 3.30 | 6.53 ± 1.13 | 1.38 ± 0.37 | 4.96 ± 0.53 | 14.89 ± 2.51 | 0.81 ± 0.51 | 2.57 ± 0.87 | 3.73 ± 0.24 |
| POX | 8.92 ± 0.01 | 5.41 ± 1.83 | 18.84 ± 6.57 | 0.82 ± 0.22 | 5.99 ± 2.90 | 0.61 ± 0.29 | 0.26 ± 0.09 | 0.71 ± 0.03 |
| OXO | 1.29 ± 0.55 | 2.28 ± 0.18 | 4.32 ± 0.98 | 4.70 ± 0.47 | 1.55 ± 0.28 | 0.61 ± 0.32 | 1.07 ± 0.32 | 0.91 ± 0.19 |
| GST | 1.19 ± 0.21 | 1.20 ± 0.26 | 1.96 ± 0.44 | 9.23 ± 0.26 | 2.27 ± 0.75 | 0.79 ± 0.29 | 0.73 ± 0.08 | 0.90 ± 0.11 |
| GLP | 0.26 ± 0.37 | 2.39 ± 0.19 | 5.26 ± 0.81 | 1.54 ± 0.53 | 2.91 ± 0.47 | 0.50 ± 0.24 | 1.04 ± 0.32 | 1.20 ± 0.18 |
| GPX | 1.04 ± 0.26 | 0.93 ± 0.14 | 0.63 ± 0.36 | 0.99 ± 0.28 | 1.55 ± 0.08 | 0.91 ± 0.37 | 3.13 ± 0.54 | 0.89 ± 0.20 |
| CAT | 16.85 ± 2.79 | 0.80 ± 0.13 | 2.22 ± 0.05 | 1.00 ± 0.27 | 1.31 ± 0.02 | 0.91 ± 0.31 | 3.93 ± 0.70 | 0.81 ± 0.29 |
| SOD | 1.05 ± 0.01 | 1.80 ± 0.19 | 0.19 ± 0.12 | 1.30 ± 0.32 | 1.07 ± 0.14 | 0.90 ± 0.12 | 0.82 ± 0.10 | 1.36 ± 0.33 |
| rpK | 16.52 ± 3.37 | 1.42 ± 0.25 | 4.02 ± 0.50 | 1.65 ± 0.23 | 0.97 ± 0.16 | 0.76 ± 0.16 | 1.37 ± 0.25 | 1.12 ± 0.27 |
| WRKY1 | 5.42 ± 4.30 | 1.30 ± 0.32 | 3.05 ± 1.04 | 2.06 ± 1.06 | 2.52 ± 0.54 | 0.75 ± 0.23 | 0.24 ± 0.05 | 1.04 ± 0.43 |
| WCK1 | 2.32 ± 1.54 | 1.25 ± 0.03 | 1.39 ± 1.36 | 1.47 ± 0.62 | 7.56 ± 0.95 | 0.66 ± 0.22 | 0.36 ± 0.17 | 0.76 ± 0.16 |

Supplementary Table 3 continued.

| Gene | PB2/MG | | | | | | | |
| --- | --- | --- | --- | --- | --- | --- | --- | --- |
|  | 6 hai | 12 hai | 24 hai | 48 hai | 3 dai | 5 dai | 9 dai | 11 dai |
| PR1 | 3.09 ± 1.10 | 5.19 ± 2.06 | 4.53 ± 1.24 | 7.66 ± 1.86 | 5.17 ± 2.18 | 1.95 ± 0.60 | 1.72 ± 0.25 | 5.02 ± 1.82 |
| CHIT | 2.37 ± 1.37 | 1.30 ± 0.37 | 0.96 ± 0.63 | 2.34 ± 0.22 | 2.01 ± 0.26 | 0.49 ± 0.23 | 0.58 ± 0.13 | 1.05 ± 0.23 |
| GLU | 2.19 ± 0.41 | 1.86 ± 0.23 | 4.95 ± 1.99 | 2.30 ± 1.1 | 22.94 ± 6.39 | 0.55 ± 0.27 | 0.44 ± 0.22 | 2.83 ± 0.82 |
| TLP | 8.69 ± 2.56 | 6.65 ± 0.69 | 0.82 ± 0.60 | 7.22 ± 2.54 | 3.09 ± 1.57 | 1.46 ± 0.27 | 1.07 ± 0.39 | 3.72 ± 0.30 |
| LIP | 0.78 ± 0.22 | 0.95 ± 0.22 | 1.32 ± 0.08 | 1.29 ± 0.10 | 5.15 ± 2.11 | 0.59 ± 0.22 | 0.86 ± 0.44 | 0.94 ± 0.27 |
| LOX | 1.13 ± 0.39 | 1.54 ± 0.35 | 0.38 ± 0.46 | 0.97 ± 0.2 | 1.06 ± 0.78 | 0.63 ± 0.37 | 0.42 ± 0.21 | 0.68 ± 0.26 |
| AOS | 1.12 ± 0.53 | 1.22 ± 0.26 | 0.87 ± 0.57 | 1.15 ± 0.35 | 13.71 ± 1.15 | 0.51 ± 0.11 | 0.31 ± 0.14 | 0.96 ± 0.41 |
| PAL | 1.35 ± 0.43 | 0.96 ± 0.35 | 2.29 ± 1.12 | 2.72 ± 0.89 | 29.54 ± 2.51 | 0.52 ± 0.11 | 0.67 ± 0.33 | 1.06 ± 0.16 |
| CHS | 0.59 ± 0.07 | 0.75 ± 0.33 | 0.72 ± 0.44 | 1.60 ± 0.32 | 1.38 ± 0.04 | 0.71 ± 0.12 | 0.67 ± 0.07 | 0.86 ± 0.29 |
| FLAV | 7.37 ± 1.63 | 4.14 ± 0.99 | 5.08 ± 0.31 | 11.5 ± 3.87 | 40.3 ± 10.23 | 1.57 ± 0.58 | 4.22 ± 0.23 | 4.76 ± 2.66 |
| POX | 2.38 ± 0.22 | 4.55 ± 0.96 | 7.35 ± 2.41 | 1.16 ± 0.27 | 11.4 ± 0.62 | 0.97 ± 0.48 | 0.30 ± 0.22 | 0.72 ± 0.31 |
| OXO | 2.22 ± 0.43 | 2.76 ± 0.94 | 3.35 ± 1.05 | 3.56 ± 0.98 | 2.15 ± 0.57 | 0.85 ± 0.18 | 1.11 ± 0.37 | 0.69 ± 0.20 |
| GST | 1.09 ± 0.38 | 0.94 ± 0.18 | 1.73 ± 0.20 | 4.47 ± 0.29 | 2.40 ± 0.30 | 0.89 ± 0.11 | 0.70 ± 0.29 | 0.74 ± 0.11 |
| GLP | 1.36 ± 0.42 | 0.92 ± 0.22 | 4.01 ± 0.58 | 1.93 ± 0.38 | 3.60 ± 0.59 | 0.71 ± 0.15 | 1.24 ± 0.33 | 0.83 ± 0.26 |
| GPX | 1.72 ± 0.14 | 0.88 ± 0.22 | 0.38 ± 0.37 | 1.10 ± 0.42 | 1.93 ± 0.19 | 1.14 ± 0.12 | 3.61 ± 0.57 | 0.87 ± 0.15 |
| CAT | 0.69 ± 0.19 | 0.83 ± 0.19 | 3.20 ± 0.62 | 0.96 ± 0.17 | 1.64 ± 0.36 | 1.23 ± 0.16 | 4.06 ± 0.70 | 1.06 ± 0.13 |
| SOD | 1.72 ± 0.14 | 1.51 ± 0.16 | 0.21 ± 0.08 | 1.09 ± 0.17 | 0.83 ± 0.17 | 0.98 ± 0.06 | 0.88 ± 0.17 | 0.85 ± 0.07 |
| rpK | 0.69 ± 0.19 | 1.10 ± 0.12 | 1.64 ± 1.39 | 1.42 ± 0.22 | 1.16 ± 0.02 | 0.96 ± 0.04 | 1.42 ± 0.22 | 0.84 ± 0.10 |
| WRKY1 | 0.92 ± 0.32 | 1.33 ± 0.17 | 7.73 ± 0.05 | 1.96 ± 0.41 | 3.22 ± 0.28 | 0.89 ± 0.14 | 0.29 ± 0.08 | 1.21 ± 0.06 |
| WCK1 | 0.70 ± 0.16 | 0.87 ± 0.21 | 0.57 ± 0.39 | 1.58 ± 0.35 | 10.27 ± 2.40 | 0.90 ± 0.20 | 0.43 ± 0.29 | 0.55 ± 0.17 |

The values shown are the mean and standard deviation of three biological replicates and five technical replicates.
